# Realizing Scalable Chemical Vapor Deposition of Monolayer Graphene Films on Iron with Concurrent Surface Hardening by *In Situ* Observations

**DOI:** 10.1021/acsami.5c18706

**Published:** 2026-01-28

**Authors:** Bernhard Fickl, Werner Artner, Daniel Matulka, Jakob Rath, Martin Nastran, Markus Hofer, Raoul Blume, Michael Hävecker, Alexander Kirnbauer, Florian Fahrnberger, Herbert Hutter, Dengsong Zhang, Paul H. Mayrhofer, Axel Knop-Gericke, Beatriz Roldan Cuenya, Robert Schlögl, Christian Dipolt, Dominik Eder, Bernhard C. Bayer

**Affiliations:** † Institute of Materials Chemistry, 164718 Technische Universität Wien (TU Wien), Getreidemarkt 9, 1060 Vienna, Austria; ‡ X-Ray Center, Technische Universität Wien (TU Wien), Getreidemarkt 9, 1060 Vienna, Austria; § Analytical Instrumentation Center (AIC), Technische Universität Wien (TU Wien), Lehargasse 6, 1060 Vienna, Austria; ∥ Max-Planck-Institut für Chemische Energiekonversion, Postfach 101365, Mülheim an der Ruhr 45413, Germany; ⊥ Department of Inorganic Chemistry, Fritz-Haber-Institut der Max-Planck Gesellschaft, Faradayweg 4-6, 14195 Berlin, Germany; # Institute of Materials Science and Technology, Technische Universität Wien (TU Wien), Getreidemarkt 9, 1060 Vienna, Austria; 7 Institute of Chemical Technologies and Analytics, Technische Universität Wien (TU Wien), Getreidemarkt 9, 1060 Vienna, Austria; 8 International Joint Laboratory of Catalytic Chemistry, State Key Laboratory of Advanced Special Steel, Innovation Institute of Carbon Neutrality, Research Center of Nanoscience and Technology, Department of Chemistry, College of Sciences, 34747 Shanghai University, No. 99 Shangda Road, Shanghai 200444, China; 9 Rübig GmbH & Co. KG, Schafwiesenstraße 56, 4600 Wels, Austria

**Keywords:** graphene, chemical vapor deposition, iron, corrosion barrier, steel

## Abstract

Graphene has been suggested as an ultimately thin functional coating for metallurgical alloys, such as steels. However, even on pure iron (Fe), the parent phase of steels, the growth of high quality graphene films remains largely elusive to date. We here report scalable chemical vapor deposition (CVD) of high quality monolayer graphene films on Fe substrates. To achieve this, we here elucidate the mechanisms of graphene growth on Fe using complementary *in situ* X-ray diffractometry (XRD) and *in situ* near ambient pressure X-ray photoelectron spectroscopy (NAP XPS) *during* our scalable CVD conditions. As key factors that set Fe apart from other common graphene CVD catalyst supports such as Ni or Cu, we identify that for Fe (i) carbothermal reduction of persistent Fe-oxides and (ii) kinetic balancing of carbon uptake into the Fe during CVD near the Fe–C eutectoid because of the complex multiphased Fe–C phase diagram are critical. Additionally, we establish that the carbon uptake into the Fe during graphene CVD is not only important in terms of growth mechanism but can also be advantageously utilized for concurrent surface hardening of the Fe during the graphene CVD process, akin to carburization/case hardening. Our work thereby forms a framework for controlled and scalable high-quality monolayer graphene film CVD on Fe including the introduction of concurrent surface hardening during graphene CVD.

## Graphical abstract

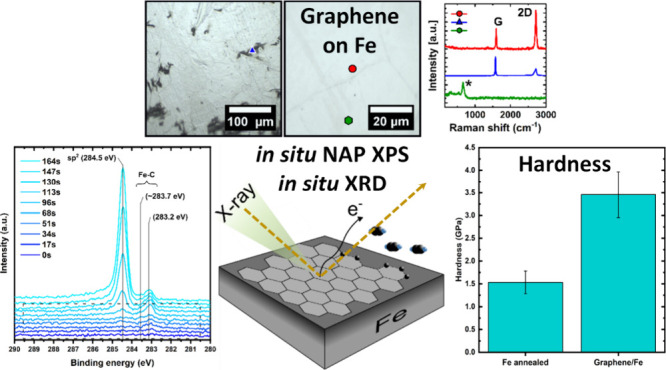

## Introduction

Two-dimensional (2D) materials, including graphene and 2D hexagonal boron nitride, have been heralded as ultimately thin functional corrosion barrier coatings for modern metallurgical alloys, including steels.^1–4^ This is because 2D materials can highly selectively impede the transport of, for example, corrosive species while enabling efficient energy/charge transfer between their substrate and the environment over ultimately small thickness scales of just one or a few atoms; an advantage which is difficult to achieve with conventional, typically much thicker (>100 nm) barrier coatings. For instance, graphene on steel can act as an effective corrosion barrier, due to its record impermeability to small molecules, while simultaneously still enabling highly efficient charge transfer between the steel and its environment, due to its high electrical conductivity, allowing for, e.g., efficient current collector/electrode functionality with ultimately minimal coating thickness. In addition, graphene coatings may offer complementary functionality such as controlled wetting, anti-icing or good biocompatibility for coatings of medical implants.^3,5^

Thus, substantial work has gone into coating metallurgical alloys and, in particular, steels with graphene as ultimately thin, functional barriers.^1–3^ Despite these efforts, however, to date, only structurally imperfect graphene coatings with incomplete coverage, high defect levels, low control over layer numbers and incomplete interfacing to the substrate have been obtained on steels, be it from top-down liquid phase exfoliation^6–12^ or bottom-up chemical vapor deposition (CVD).^13–20^ Importantly, even on pure iron (Fe), the parent phase for all steels, there have been practically no reports of monolayered graphene films with good quality and complete coverage, let alone under scalable conditions.^21–35^ This lack of graphene growth on even simple, pure Fe is thereby a clear hindrance to further advancing graphene growth on more complex, multielement, multiphased steels.

Filling this critical gap, we report here the scalable CVD of monolayered graphene films on Fe substrates. Importantly, our here reported CVD conditions are scalable and compatible with current gas phase surface hardening/carburization processes as used in the metallurgical industry. Consequently, we also demonstrate that our graphene CVD process leads to concurrent surface hardening of the Fe substrates via carbon uptake into the Fe subsurface and bulk. To achieve this goal of monolayer graphene film CVD on Fe, we here also elucidate the mechanisms of graphene growth on Fe using complementary *in situ* X-ray diffractometry (XRD) and *in situ* near ambient pressure (NAP) X-ray photoelectron spectroscopy (XPS) *during* our scalable CVD conditions to understand the complex interplay of the Fe’s surface, subsurface and bulk with the gaseous hydrocarbon CVD precursors and residual trace gases under kinetically controlled CVD process conditions. Through our *in situ* characterization-guided CVD, we thereby form a holistic framework for the process development of controlled and scalable high-quality monolayer graphene CVD on Fe-type substrates, including the introduction of concurrent surface hardening, which we expect to also lay the basis for subsequent, future expansion of graphene CVD coatings on persistently challenging steel substrates.

Graphene CVD generally relies on the catalytic decomposition of gaseous hydrocarbons at elevated temperatures (∼400–1000 °C) on metal substrates.^36^ As prior work has shown,^37–39^ unlike conventional CVD of classical μm-thick coatings, where the substrate is comparatively “inert”, in graphene CVD, the growth substrate has a highly active catalytic role via surface catalytic activity and also bulk solubilities/diffusivities.^36,40^ In particular, the substantial uptake of carbon, graphene’s constituent element, into the growth substrate’s bulk can occur during graphene CVD. This complicates graphene growth kinetics and requires close matching of CVD conditions (temperature profiles, precursor fluxes, etc.) with the growth substrate. In the past, graphene CVD has been optimized for dedicated, often sacrificial, high-purity Cu and Ni metal growth catalyst supports, achieving fully covering, layer-number-controlled, high-quality graphene films.^37–39,41–43^ In comparison, graphene CVD on Fe has been significantly lagging behind.^21–35^

This limited progress in Fe arises from several substrate-specific challenges. First, the complex Fe–C phase diagram (Figure 1) includes multiple temperature-dependent phase transitions, most importantly, the transition from body-centered-cubic (bcc) to face-centered-cubic (fcc) at the eutectoid above 727 °C, resulting in a large increase in carbon solubility.^44,45^ Such high, temperature-dependent solubility often favors 2D material growth from precipitation and thus typically yields 2D films of low quality when Fe-based supports are used.^42,46^ As we demonstrate in this report, the key to overcoming this limitation is the identification of kinetic conditions for CVD in terms of temperatures, precursor concentrations, and diffusion fluxes to facilitate predominantly isothermal growth on Fe.

**Figure 1:**
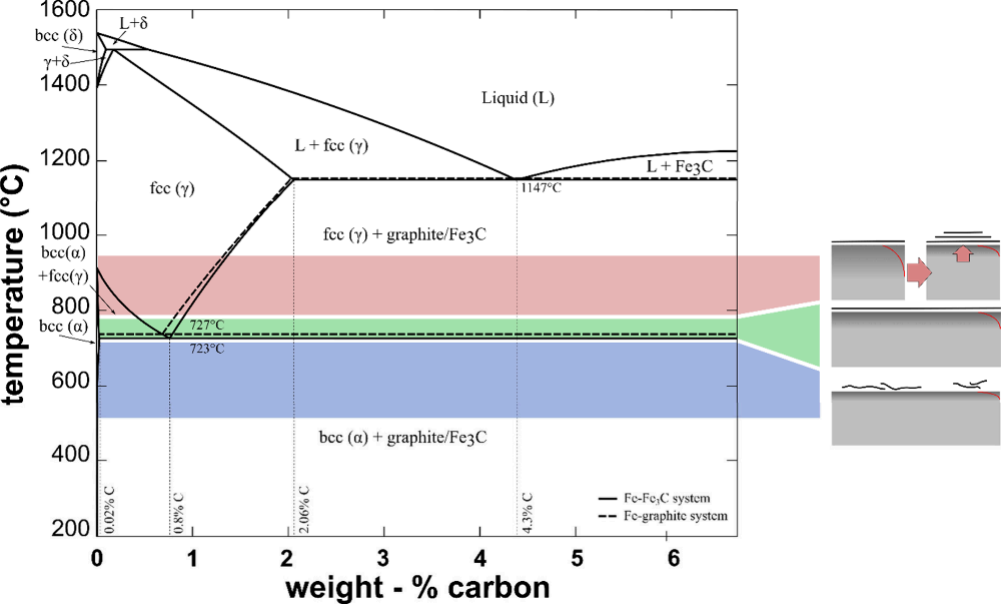
Schematic illustration of the Fe–C phase diagram for both Fe-graphite (dashed) and (metastable) Fe–Fe_3_C systems (adapted from OpenCalphad^54^ and modified), with a schematic illustration of the main findings of this study regarding the interplay of CVD conditions and graphene growth results superimposed (see Discussion section). The metastable intermetallic Fe_3_C phase is at 6.67 wt % C (at the right end of this diagram).

Second, Fe-based catalysts are set apart from the widely used Ni and Cu graphene growth catalyst supports, due to their propensity to readily form persistent surface oxides during Fe storage in ambient conditions before CVD and also from residual oxidizing species (trace oxygen and water) *in situ* during CVD. Surface oxides typically suppress Fe catalytic ability for graphene CVD entirely or, at best, only lead to defective graphene.^47^ Therefore, a reduction step with reductive gases, e.g., annealing in H_2_, is typically employed before hydrocarbon exposure in graphene CVD, and a reductive gas is also typically added during the hydrocarbon exposure to suppress *in situ* oxidation. In comparison to Ni and Cu support, we show here, however, that typical reduction conditions with H_2_ are insufficient for Fe reduction under scalable CVD conditions but that also carbothermal reduction of Fe-oxides from hydrocarbon exposure is a key element in monolayer graphene CVD on Fe. This highlights that not only the kinetics for graphene growth (as eluded to above) but also the kinetics for reduction of persistent Fe-oxides must be controlled for graphene CVD development on Fe.

Third, as mentioned previously, an aspect that is of particular usefulness for Fe is that the carbon uptake into the subsurface and bulk during graphene CVD (which we also evidence using *in situ* XRD and XPS) is reminiscent of industrially widely applied carburization hardening (case hardening) for Fe/steels.^48^ Exploring this aspect, we finally also demonstrate that under our optimized graphene CVD growth conditions, the remaining significant carbon uptake into the Fe bulk also leads to concurrent surface hardening of the Fe substrates. Thus, from a metallurgical application perspective, a beneficial interplay of concurrent graphene CVD and surface hardening is demonstrated here.

To elaborate further, previous *in situ* investigations on Ni and Cu catalyst substrates have revealed that graphene CVD follows a bulk-mediated surface growth mechanism.^37–39^ This implies that graphene CVD is governed both by surface processes (gaseous precursor breakdown and reorganization of surface species into graphene nuclei/domains) and bulk mediation, in which precursor supersaturation by diffusion on the surface, into the subsurface and, depending on kinetics, also into the bulk of the support, must be reached before graphene nucleation/growth can occur.^36–40^ Growth can then proceed isothermally on the surface and/or via precipitation from the bulk upon cooling.^37–39^ Importantly, for a given catalyst support with a given C solubility, the exact pathways of graphene CVD within this interplay of surface processes and bulk mediation can be kinetically controlled.^36,40^ The key hereby is controlling the balance between incoming precursor flux, flux to the graphene’s growth front, and the flux diffusing into the catalyst support bulk. In general, isothermal surface growth typically leads to better control over 2D materials layer numbers, quality, and coverage, while precipitation upon cooling typically leads to undefined growth with inhomogeneous layer numbers and coverage and poorer crystalline quality when using gaseous precursors and standard CVD methods.^42,49^ While previous works have demonstrated that intrinsic carbon in Cu substrate foils substantially affects graphene CVD growth,^50,51^ and graphene growth on copper requires a carbon supersaturation at the substrate (sub)­surface prior to growth,^52^ the inherently low carbon solubility of copper makes it fundamentally different from graphene growth on iron.^39,53^ Additionally, unlike Ni and Cu catalyst supports, which were shown to remain single-phased during the entire graphene CVD process,^37–39^ we explicitly show here that the Fe catalyst can undergo substantial phase transitions that are both temperature and process-stage-dependent.

## Results

### Rationally Designed CVD Conditions

To ensure fine control over the carbon flux, we base our CVD recipe on C_2_H_2_ as the hydrocarbon source. The investigated process parameters are initially based on prior developed CVD conditions for Ni catalyst supports.^37,38^ C_2_H_2_ has the advantage of dissociating readily and being active for graphene growth already at lower temperatures from ∼450 °C.^37^ Thus, C_2_H_2_ can be employed at low and well-controlled fluxes for graphene CVD. We here employ the C_2_H_2_ in a simple custom-built hot-wall quartz tube furnace with mass-flow-controlled C_2_H_2_ flux under medium-pressure CVD conditions obtained by a simple pump setup (base pressure 3 × 10^–3^ mbar). We used H_2_ as a reductive gas for pre-CVD reduction annealing and also during hydrocarbon exposure. In the CVD process, samples are heated in ∼1 mbar of H_2_ (250 sccm flow) to their target temperatures of 500 to 800 °C at ∼100 °C/min heating rate and, upon reaching the desired temperature, undergo an annealing step in 1 mbar of H_2_ (250 sccm flow) for 30 min. Then, 0.1 to 10 sccm C_2_H_2_ are added to the H_2_ (250 sccm) for the growth step for another 30 min. Subsequently, C_2_H_2_ and the heater are switched off simultaneously, and samples are left to cool naturally in ∼1 mbar of H_2_ (natural cooling at ∼35 °C/min to ∼300 °C, then ∼15 min to room temperature; split tube furnace around quartz tube opened during cooling). We emphasize that such CVD conditions are directly compatible with common carburization hardening conditions in industrial surface hardening processes^48^ and are thus intrinsically industrially scalable. High-purity 100 μm thick Fe foils (Alfa Aesar Puratonic, 99.995%) were used as a growth substrate. We deliberately chose the comparatively high thickness of the Fe foils to also account for bulk effects that have been shown to play an important role for Ni catalysts.^36,40^ For further information on experimental details, see the Methods section.

### Optimization of Graphene CVD Results

We first describe a survey of the CVD parameter space to illustrate our optimized growth results before providing experimental (*in situ*) insights into the corresponding growth mechanisms further below. Figure 2 shows optical microscopy images (left) and corresponding spot-localized Raman spectra (right, spot localization indicated by colored spectra/spots) of growth results on the Fe supports from the above describe CVD conditions for an intermediate C_2_H_2_ flux of 1 sccm as a function of growth temperature from 500 to 800 °C (and referenced against as-received Fe foil). For the as-received Fe foil, we find in optical microscopy and Raman (green trace) that the foils have formed surface Fe-oxides from storage in ambient air.^55,56^ The substrate surface is homogeneous on the macroscopic scale and the optical images are therefore representative of the whole ∼1 cm × 1 cm substrate foil surface. After 500 °C CVD, we find the Fe foil to be inhomogeneously covered by nanocrystalline graphite (red trace: intensity ratio D/G > 2 and very low 2D intensity^57,58^ ) and amorphous carbon (blue trace: merged D and G, no 2D^57,58^ ) regions. Under these nanocrystalline graphene and amorphous carbon regions, no signs of remaining Fe-oxide are detected in Raman, implying localized reduction of the Fe-oxides during the CVD process. The graphitization level of the carbon deposits from the 500 °C growth temperature indicates insufficient thermal activation for healing out defects in the growing carbon film.^59^ With increasing temperature to 600 °C, we accordingly find an improvement in graphitization levels: We grow inhomogeneous multilayer graphene films at 600 °C without (blue trace: intensity ratio D/G ≈ 0.3; 2D/G ≈ 0.7)^58^ and with remaining Fe-oxides (red trace) and small graphene-bare Fe-oxide regions (green trace). When further increasing the growth temperature to 700 °C, we find further improvements in graphitization, indicated by a further reduction in D/G ratio to <0.2. Additionally, we now find an inhomogeneous mixture of multilayer graphene (blue trace) as well as monolayer graphene regions (red trace: 2D/G ∼ 1.5).^58^ Notably, however, persistent Fe-oxides are still detected (green trace). Further increasing the growth temperature to 750 °C we find clear improvements in homogeneity, importantly toward predominantly monolayer graphene growth of high quality (red trace: D/G < 0.05; 2D/G ≈ 2).^58^ Quantitatively, we estimate monolayer graphene sample coverage to be ∼70% (based on optical micrographs and Raman analysis). The remaining nonmonolayer-graphene areas are comprised of isolated multilayer graphene islands to ∼10% sample coverage (dark spots in leftmost optical micrograph, blue trace) and remaining Fe-oxide regions (∼20%), which are, however, void of graphene or carbon coverage (green trace). Thereby, monolayer and multilayer graphene regions combined have a coverage of ∼80% on the iron. We further confirm our monolayer assignment of these graphene films (and exclude formation of turbostratic graphite) via a standard polymer-assisted transfer^60^ of the films from their Fe support onto 90 nm SiO_2_-coated Si wafers and further Raman and optical microscopy data in Figures S1 and S2. Interestingly, when further increasing the CVD temperature to 800 °C, we do *not* observe further improvements in controlled graphene coverage but instead obtain comparatively much more inhomogeneous carbon films with only a small fraction of monolayer graphene coverage (red trace) but large fractions of multilayer graphene growth (dark patches in the leftmost image, blue trace) as well as bare remaining Fe-oxide regions (green trace). Notably, however, growth at 800 °C retains similarly high graphitic quality^58^ (D/G < 0.05) as for 750 °C. This indicates that at 800 °C, not graphitization but other growth mechanistic factors prevent predominant monolayer graphene film growth. This observation is consistent with previous reports, who found that thin graphene layers can be synthesized at temperatures below 1000 °C, while higher-temperature CVD growth (≥1100 °C), leads to the formation of thicker multilayer or graphitic carbon structures due to increased carbon dissolution and precipitation upon cooling.^21,35,61^

**Figure 2:**
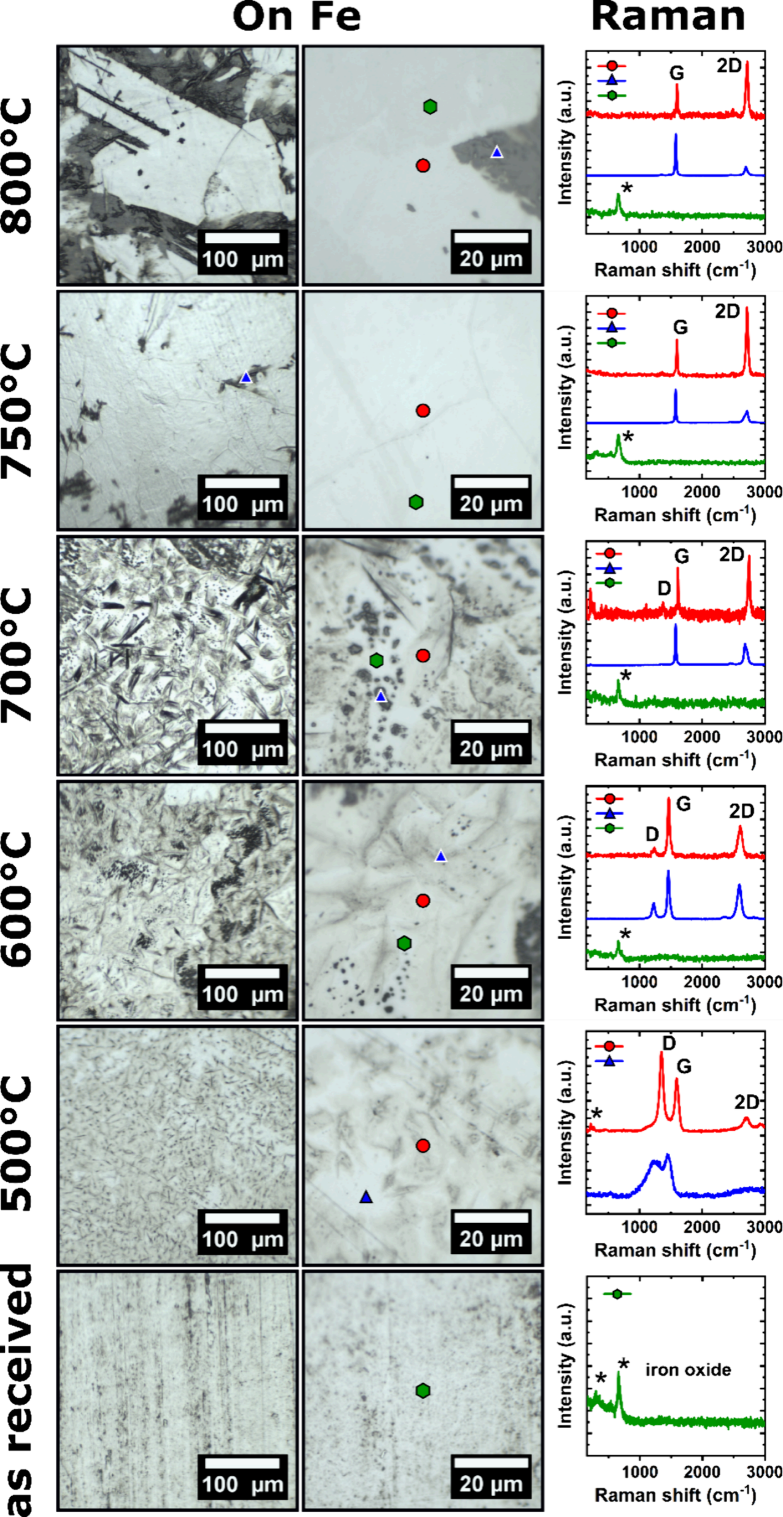
Optical micrographs (left and middle panels) at different magnifications and spot-localized Raman spectra (right panel, spot localization indicated by colored spectra/spots) of as received 100 μm Fe foils and growth results on Fe after CVD with 1 sccm C_2_H_2_ at temperatures from 500 to 800 °C. Carbon related D, G, and 2D Raman peaks^58^ are labeled and iron oxide related peaks^55,56^ are indicated by “*” in the Raman spectra.

Taking the so far best monolayer graphene results from CVD at 750 °C at 1 sccm C_2_H_2_ from Figure 2 as an optimized reference point, we then compare the effect of C_2_H_2_ flux in Figure S3. We find that growth at a lower C_2_H_2_ flux of ∼0.1 sccm leads to only monolayer island growth with large areas of the substrate left covered in Fe-oxide. This indicates an insufficient carbon flux. Conversely, growth at an increased 10 sccm C_2_H_2_ flux leads to a relative increase in large area multilayer graphitic growth, thus implying that 10 sccm C_2_H_2_ represent a too high carbon flux for predominant monolayer growth. This suggests that overall at 750 °C, the 1 sccm C_2_H_2_ flux represents, under the screened conditions, an optimized balance of incoming precursor carbon flux, carbon flux to the graphene’s growth front and what carbon flux is diffused into the catalyst support bulk toward best monolayer graphene growth results.^36^ To assess the evolution of monolayer graphene coverage and domain size for the optimized 1 sccm C_2_H_2_ flux, we reduce hydrocarbon exposure times to 15 and 5 min. As shown in Figure S4, this reduction leads to a decrease in monolayer coverage from approximately 70% at 30 min to ∼50% at 15 min and ∼20% at 5 min. Concurrently, the multilayer fraction also declines with shorter exposure times, though to a lesser extent, dropping from ∼12% at 30 min, to ∼7% at 15 min, and ∼5% at 5 min. Notably, graphene domains begin to coalesce even at the shortest exposure time, as is evident in Figure S4. Extending the deposition time to 60 min, as shown in Figure S5, results in a modest increase in the multilayer fraction from ∼10% to ∼18%, while the overall coverage remains unchanged. This indicates that longer deposition times under our current conditions do not enhance monolayer coverage.

We note that overall, our optimized results here at 750 °C in Figure 2 go beyond prior graphene CVD on Fe in terms of quality and monolayer coverage, particularly under scalable CVD conditions.^21–35^

### Investigation of Growth Mechanisms

After having established our optimized graphene CVD protocol on Fe, we now turn to elucidating the underlying mechanisms, including *in situ* investigations. We first investigate the key importance of, as we find, carbothermal surface Fe-oxide reduction during CVD growth, before investigating the Fe–C phase and surface chemistry evolution in/on the Fe catalyst support foils during our optimized CVD conditions by complementary *in situ* XRD and *in situ* NAP XPS.

### Importance of Carbothermal Reduction of Fe-Oxides

Our data in Figure 2 indicates that the presence of persistent surface Fe-oxides, which we detect as a minority surface coverage under practically all CVD conditions tested, is a remaining unfavorable factor in our graphene growth on Fe. Such Fe-oxides are detrimental to graphene growth because generally oxides are known to be much less suited to catalyze high-quality graphene during CVD.^47,62^ Surface Fe-oxides can form from ambient air during Fe catalyst support storage prior to CVD, including subsequent Fe-oxide crystallization during the high temperature CVD process and/or from *in situ* oxidation of the Fe catalyst support from residual trace gases such as O_2_ or water during the CVD process.^63^ To counter both processes and reduce such Fe-surface oxides, most CVD recipes, including ours, use a dedicated reductive pretreatment step (here 1 mbar H_2_) and/or a reductive ad-gas (here 1 mbar H_2_) being present throughout the entire CVD process. Additionally, however, carbothermal reduction of the Fe-oxides from the hydrocarbon source (here C_2_H_2_) may also occur but is commonly not explicitly considered. Compared to other established graphene catalysts, Fe-oxides are, however, known to be more stable and intrinsically harder to reduce than, in comparison, under their respective CVD conditions, readily reducible Ni-oxides^37,38^ and Cu-oxides.^39^

To therefore disentangle Fe-oxide formation and reduction processes under our CVD conditions, we conducted cross-check experiments. In Figure S6, we present optical microscopy and Raman spectroscopy results for 100 μm Fe foils that underwent the CVD process at 750 °C but *without* C_2_H_2_ addition, i.e., samples only underwent annealing in H_2_. For these samples, we find no graphene growth (as expected due to no C_2_H_2_ exposure); however, despite the reducing H_2_ conditions, the presence of a surface Fe-oxide over the entire Fe foil surface is detected. Together with the observed presence of an initial surface Fe-oxide in our as received foils (Figure 2), this implies that under our conditions (and in our CVD furnace) the H_2_ alone is not sufficient for initially present Fe-oxide reduction and suggests that the C_2_H_2_ under our process conditions has not only the role of graphene growth precursor but also of a carbothermal reduction agent, since only with C_2_H_2_ introduction as in Figure 2 the majority of the Fe has been reduced (as indirectly evidenced by the observed carbon growth). Our results in Figure 2 nevertheless show that through carbothermal reduction, good graphene films can already be achieved on Fe by simple and well-scalable medium vacuum conditions (∼10^–3^ mbar base pressure), as employed here.

### Fe–C Phase Dynamics during CVD by *In Situ* XRD

After having *ex situ* investigated the importance of enabling surface Fe-oxide reduction, including carbothermal reduction, we now turn to investigate the Fe–C phase dynamics during graphene CVD on Fe catalyst supports. Figure S7 shows *ex situ* XRD patterns of the Fe supports before and after CVD processing corresponding to Figure 2. As-received foils are, at room temperature, of phase-pure metallic body-centered-cubic (bcc) Fe (α-Fe) structure in accordance with the phase diagram (Figure 1). No Fe-oxides are detected in the XRD, further confirming that the Fe-oxides in Raman (Figure 2) are surface oxides. After CVD and subsequent cooling to room temperature, we find for all growth temperatures the majority phase to be bcc-Fe but a minority Fe-carbide Fe_3_C phase has been formed additionally during CVD. (Note the square-root intensity scale in Figure S7 that strongly emphasizes this minority Fe_3_C phase. Rietveld refinement puts a maximum phase contribution of Fe_3_C to ∼12%.) A graphite-related (002) peak is detected as a function of growth temperature in accordance with the presence and roughly the amount of multilayer graphene compared to Figure 2. The observation of a Fe-carbide signal in Figure S7 implies that during the CVD process, the Fe catalyst support is subjected to an influx of carbon into the catalyst bulk, resulting in the observed formation of an additional Fe_3_C phase. To investigate this phase evolution further, we therefore turn, in Figure 3, to process-step-resolved *in situ* XRD measurements during our optimized CVD conditions at ∼750 °C to reveal the phase evolution of the Fe catalyst support *during* each CVD process step. Here we find that the initial bcc Fe retains its bcc Fe structure during the H_2_ annealing step at 750 °C, but during the subsequent C_2_H_2_ exposure at 750 °C undergoes a phase transition to the face-centered-cubic (fcc) Fe (γ-Fe) phase. This is direct evidence for the carbon influx into the catalyst bulk during the C_2_H_2_ exposure, because according to the phase diagram (Figure 1) with increasing carbon concentration in the Fe a phase transition from bcc to fcc Fe occurs for growth temperatures above the eutectoid temperature of ∼723 °C.^44,45^ Concurrently we observe the emergence of a graphite peak during C_2_H_2_ exposure, giving direct evidence of isothermal graphene growth via our *in situ* XRD experiments. The observation of fcc Fe as the predominant phase during graphene CVD reaffirms that *ex situ* XRD measurements such as in Figure S7 can not necessarily capture the relevant phase evolution (as no fcc Fe has been detected in Figure S7 at all) but that *in situ* experiments are necessary.^44,45^ After CVD and cooling to room temperature, we observe that the Fe has reverted back to bcc Fe (again in agreement with the phase diagram in Figure 1). During our *in situ* XRD measurements, no indication of substantial Fe_3_C formation during the CVD process was observed. We note, however, that in our *in situ* XRD runs in Figure 3, we employed a Cu anode, which for Fe samples results in a higher background due to fluorescence, while our *ex situ* XRD data in Figure S7 was measured with a Cr anode that allows for higher sensitivity.^64^ Conversely, when *ex situ* remeasuring our *in situ* sample from Figure 3 with a Cr anode after CVD we accordingly measure a very small Fe_3_C signal, which could have formed either during C_2_H_2_ exposure or during cooling (top pattern in Figure 3). (Note the square-root intensity scale in Figure 3. Rietveld refinement puts Fe_3_C to an upper limit of ∼12%, fully consistent with the *ex situ* growth in Figure S7.) Combined, our *in situ* XRD data at optimized CVD conditions therefore indicates that fcc Fe is the majority phase in the Fe foils during growth and that a minority Fe_3_C phase could possibly also be present during growth. In either case, the above XRD data confirm the carbon uptake into Fe as an important factor during growth (which results in the bcc to fcc Fe transition and Fe_3_C formation) and that graphene growth occurs (at least partially) isothermally. The time resolution of our *in situ* XRD measurements is, however, not sufficient to disentangle the dynamics of the isothermal graphene growth and answer whether growth via precipitation of prior dissolved carbon during cooling also contributes to graphene growth. To answer these questions, we turned to *in situ* NAP XPS with better time resolution.

**Figure 3:**
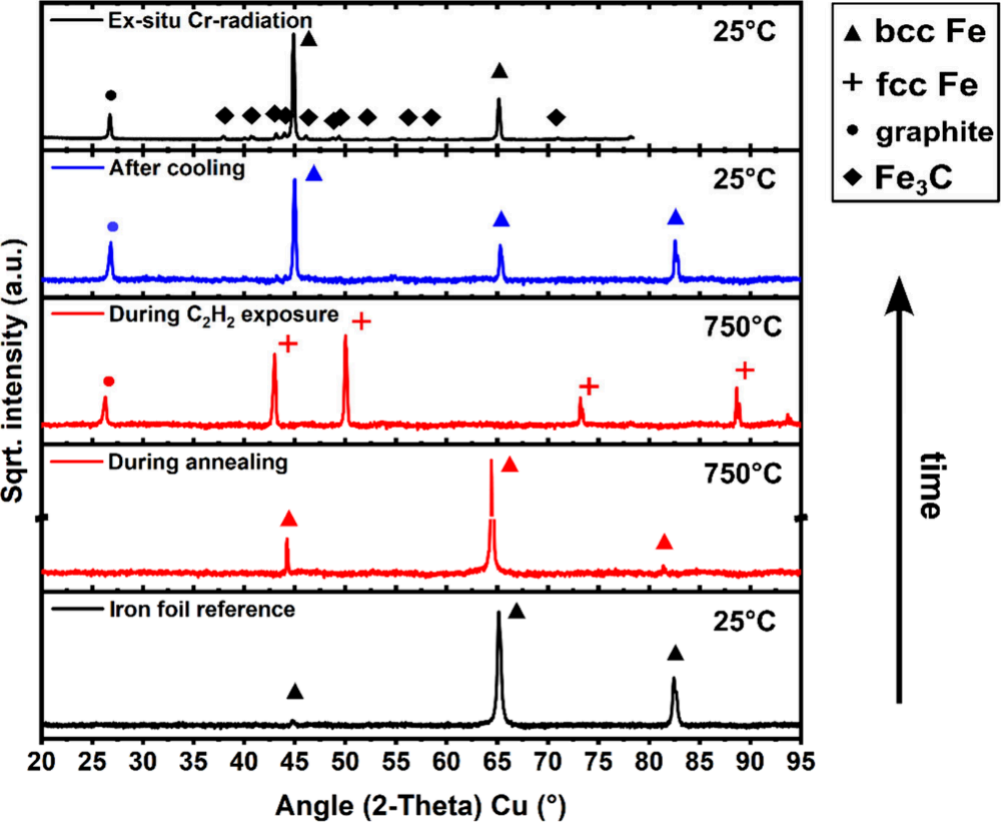
Process-step resolved *in situ* XRD patterns of Fe catalyst during CVD at ∼750 °C. Process step conditions (from bottom to top) are indicated. Salient phases identified are indicated (International Centre for Diffraction Data (ICDD), PDF-5+ database, powder diffraction file entry: bcc-Fe-ambient 04-015-8438; bcc-Fe-nonambient 040-17-5839; fcc-Fe-nonambient 04-003-1443; carbon/graphite 04-016-0554; Fe_3_C 04-007-0422). The *in situ* XRD patterns were measured with a Cu anode, resulting in higher background signal for Fe,^64^ while the uppermost pattern was measured *ex situ* after CVD with a Cr anode thus also detecting a minority Fe_3_C phase that was below the sensitivity of the *in situ* Cu anode measurements (Cr anode pattern recalculated to 2θ angles comparable to Cu anode data set). Note that the intensity scale is plotted in square-root and with intensity scale interruption(s) for better visualization of minority phase signals. Cr anode 2θ data was recalculated to the Cu anode for better comparison. In Figure S8, we show the substrate surface after our *in situ* XRD process, through optical microscopy and Raman spectroscopy. While the amount of multilayer graphitic regions is higher in comparison to our optimized *ex situ* process in Figure 2, we nevertheless find regions of similar graphene quality and overall similar growth morphologies. Slight differences in growth outcome between *ex situ* and *in situ* routes are typical for multitechnique *in situ* studies in graphene CVD.^37–39,41^ We note that the change of heating (XRD heating chamber versus split tube furnace), pumping (different vacuum system and pumps), and gas delivery can affect the growth behavior. Under these aspects, the still generally similar growth outcome after our *in situ* XRD process is a good indication of the still good enough comparability of the two process environments.

### Surface Evolution during CVD by *In Situ* NAP XPS

We employ *in situ* NAP XPS to study the surface evolution of carbon and Fe and their interactions throughout the graphene growth process at the same nominal conditions as in our optimized growth from Figure 2. Notably, while we investigate the bulk of the Fe sample volume in our XRD measurements in Figure 3 (and at only tens of minutes time resolution), with the XPS measurements, we probe the uppermost few nm of the sample surface and subsurface and at a time resolution of seconds.^43^

Figure 4a,b shows time-resolved C 1s spectral evolution during the C_2_H_2_ exposure step at 750 °C. The Fe sample is initially fully clean from adventitious carbon (removed during the H_2_ pretreatment) as evidenced by the flat C1st spectrum at 0 s in Figure 4a,b. Upon C_2_H_2_ exposure, we first observed the emergence of a peak at 283.2 eV, starting at ∼17 s. This 283.2 eV peak we ascribe to carbon bonded at iron surface sites based on previous work using Ni substrates.^38,65^ This component also has an asymmetric shoulder toward higher binding energies at 283.7 eV (see in particular Figure 4b), which becomes more visible with time. This shoulder can be ascribed to an additional C 1s component at 283.7 eV, which we attribute to carbon dissolved in Fe, again based on prior work.^38,65^ Both the 283.2 eV component and 283.7 eV shoulder are thereby direct signs of carbon influx into the Fe, in excellent accordance with the XRD data above. We label both 283.2 and 283.7 eV components, therefore, as “Fe–C”. Notably, both Fe–C components (283.2 and 283.7 eV) precede the first emergence of the C 1s component of sp^2^ graphene at 284.5 eV, which emerges only after an incubation time of ∼51 s at 750 °C after C_2_H_2_ introduction. Thereby, the Fe–C 283.2 and 283.7 eV components are indicative of the necessary carbon influx into the Fe subsurface *before* graphene nucleation can occur. After the first emergence of the sp^2^ graphene signal at 284.5 eV after ∼51 s, the graphene sp^2^ signal overtakes the Fe–C components in intensity after ∼113 s and then continues to rise with an increasing C_2_H_2_ exposure time. This is further direct evidence of isothermal graphene growth on the Fe. (In Figure S9 we show detailed C 1s components fits to the experimental XPS data.)

**Figure 4:**
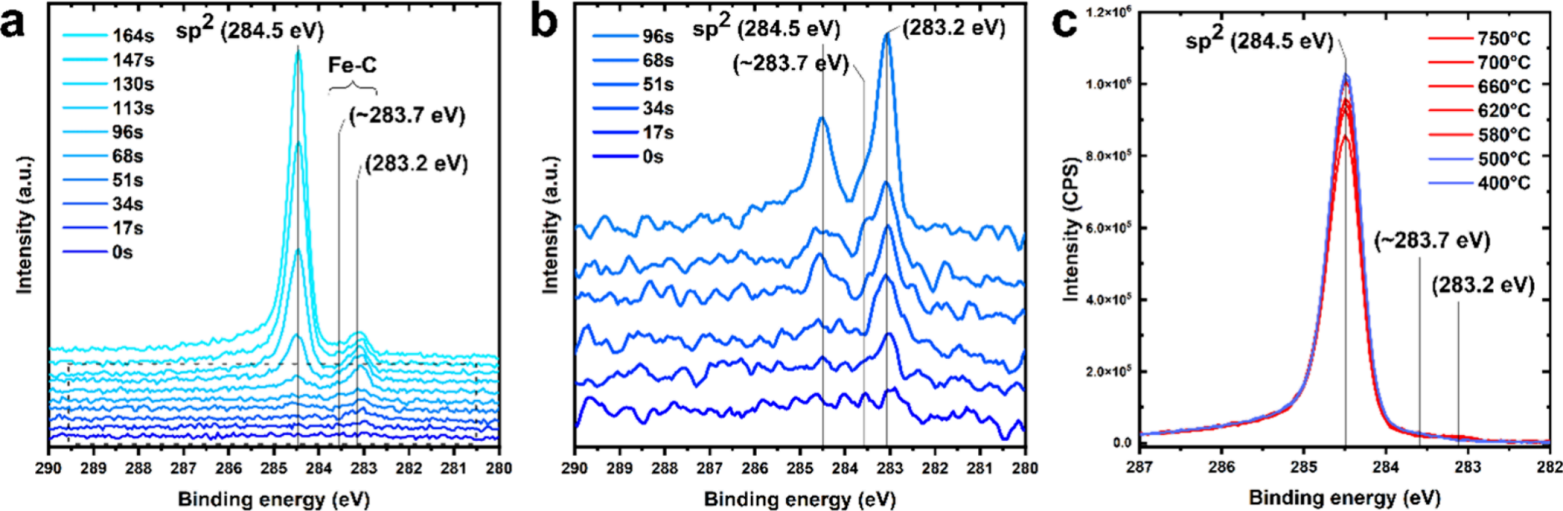
(a) C 1s time-resolved in situ NAP XPS spectra during C_2_H_2_ exposure at 750 °C. Salient C 1s components are indicated. (b) Zoom-in on the corresponding region in panel (a). (c) C 1s time-resolved after C_2_H_2_ shut off during cooling in H_2_ from 750 °C. A fit to the XPS data is shown in Figure S9, showing consistency with high-quality graphene, reaffirming the Raman analysis in Figures S1 and S2.^39,66^ Figure S10 presents excellent agreement in optical microscopy and Raman results to the *ex situ* growth in Figure 2, fully justifying the comparability of our *in situ* studies with *ex situ* CVD results.

To resolve if carbon precipitation upon cooling also contributes to graphene growth from Fe under our optimized conditions, we also follow the C 1s evolution after C_2_H_2_ shut off during the cooling step (∼50 °C/min in H_2_) in a time-resolved fashion in Figure 4c. We find only a small increase in the graphene C 1s signal at 284.5 eV during cooling (by ∼18%), showing thereby that under our growth conditions, additional graphene formation by precipitation of prior dissolved carbon from Fe upon temperature cooling is limited. This links excellently with the observed only minor multilayer graphene coverage in Figure 2 at optimized monolayer graphene growth conditions at 750 °C.

The *in situ* NAP XPS data thereby indicate that the growth kinetics for our 750 °C growth on Fe are well controlled toward almost exclusive isothermal graphene growth with minimal additional graphene growth by precipitation upon cooling. In line with the XRD data, also recorded for these kinetic conditions, a significant carbon uptake into the Fe subsurface (and from XRD Fe bulk) is evidenced as part of the graphene growth process. While this carbon reservoir is crucial to the isothermal graphene growth process at 750 °C, it contributes only slightly to graphene growth via minor precipitation upon cooling.

A corollary to this finding is that when we increase the Fe “reservoir” for carbon uptake into Fe with otherwise similar carbon feeding flux, we should change the growth kinetics toward a higher contribution of precipitation upon cooling growth. We test this hypothesis by measuring *in situ* NAP XPS also during higher temperature exposure at 800 °C. Based on the phase diagram at 800 °C (Figure 1) we would expect a higher carbon solubility in Fe and thus a larger free “reservoir” to accommodate carbon atoms in the Fe at the higher temperature. This should, for instance, directly translate to a longer filling period of this “reservoir” and thus a longer carbon uptake period before graphene nucleation. This is because the prefilling of the “reservoir” competes with reaching the surface carbon supersaturation necessary for isothermal graphene nucleation and growth. Following this line of argument, when looking at the *in situ* C 1s data in Figure S11a, we indeed find that the 800 °C growth temperature leads to an increased incubation period of ∼10 min (i.e., ∼10 times longer than the ∼51 s observed at 750 °C). During this period, only the Fe–C components (283.2 and 283.7 eV) are visible before the graphene sp^2^ signal at 284.5 eV appears and graphene isothermally grows. Consistently, Figure S11b shows that, after C_2_H_2_ exposure and subsequent cooling from 800 °C, the graphene C 1s signal at 284.5 eV increases by 64%, compared to only 18% at 750 °C. This larger increase confirms that precipitation during cooling contributes more significantly to the overall growth at 800 °C. This is in excellent agreement with the increased multilayer fraction for the growth results at 800 °C compared to the optimized 750 °C, visible in the *ex situ* data in Figure 2. We thus confirm that at elevated growth temperatures (≥800 °C), the increased carbon solubility in Fe and enhanced bulk diffusion rates promote excessive carbon uptake into the substrate, which, upon cooling, results in carbon precipitation and uncontrolled multilayer formation. This transition from kinetically limited surface-mediated growth to precipitation-driven growth explains the diminishing monolayer selectivity at higher temperatures. Even higher temperatures have been accordingly shown to produce thick multilayer carbon structures by precipitation upon cooling.^61^

### Concurrent Surface Hardening during Graphene CVD

After having established the key role of carbon influx into the Fe subsurface and bulk during the graphene CVD process via our (*in situ*) observations above, we finally probed the technological implications of this carbon influx. In particular, the clearly observed carbon uptake into Fe is highly reminiscent of industrially widely applied carburization hardening (case hardening) processes for Fe/steels.^48^ Comparing a Fe sample that underwent optimized graphene CVD at 750 °C with a sample that underwent similar H_2_ annealing at 750 °C but without the C_2_H_2_ exposure step (i.e., no graphene growth), we show in Figure S12 using depth-profiling of the carbon signal via time-of-flight secondary-ion-mass-spectrometry (ToF-SIMS), that significant carbon uptake into the Fe bulk from the C_2_H_2_ exposure has taken place (at least to ∼1 μm depth) compared to a practically carbon-free, only H_2_ annealed Fe reference sample. This *ex situ* data is thereby in excellent agreement with the (*in situ*) XRD and XPS data above.

Consequently, we finally tested the effect of this carbon uptake on Fe surface hardness. Employing nanoindentation measurements (Figure 5), we obtain hardness values for a graphene/Fe sample after optimized graphene CVD at 750 °C, compared to an Fe reference sample annealed only in H_2_. The data in Figure 5 clearly show a drastic increase in hardness by ∼130% that results from the carbon influx during graphene CVD. We thereby establish that surface hardening occurs concurrently with the graphene CVD process. The above observed substantial carbon enrichment relates to classic carburization-type hardening, where interstitial carbon atoms distort the Fe lattice and act as obstacles to dislocation motion.^48^ In addition, our XRD and XPS data confirm the formation of iron carbides, which further contributes to the observed hardening by introducing additional barriers to deformation. Notably, these beneficial hardening mechanisms arise here inherently as a byproduct of the catalytic graphene CVD environment.

**Figure 5:**
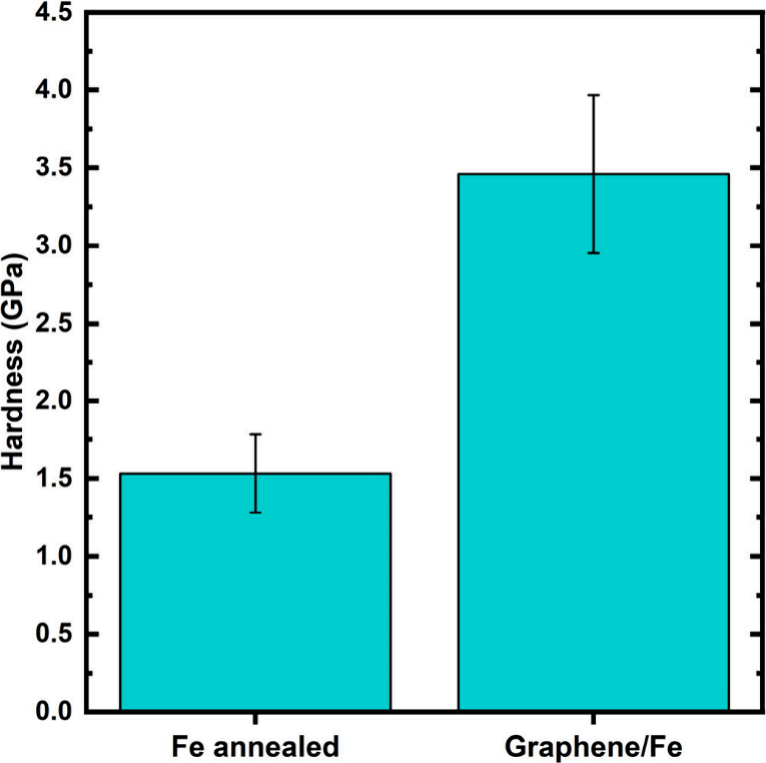
Hardness values from nanoindentation experiments for a graphene/Fe sample after optimized graphene CVD at 750 °C (1 sccm C_2_H_2_, Figure 2) against an only H_2_-annealed Fe reference sample (i.e., without C_2_H_2_ exposure), elucidating a surface hardening effect concurrent to graphene growth under our optimized CVD conditions.

## Discussion

This study provides a general framework for optimizing graphene CVD on Fe by controlling the temperature-dependent kinetics of the Fe–C system. We illustrate our findings as schematic sketches of the Fe–C phase diagram in Figure 1, showing that a narrow temperature window around 750 °C, just above the eutectoid, emerges as ideal for predominantly isothermal graphene growth on Fe, enabling high-quality monolayer graphene formation with minimal precipitation-induced multilayers upon cooling.

For growth temperatures well below the Fe–C eutectoid (∼723 °C), graphene quality/crystallinity is restricted by insufficient energy, while at higher temperatures (≥800 °C), excessive carbon uptake into the Fe bulk results in a larger area of multilayers through carbon precipitation upon cooling. These insights highlight the importance of finding a kinetic balance for graphene growth on Fe for recipe development. We expect the above rationale to be applicable as a general guideline for the Fe–C system, albeit particular conditions will need adjustments for, e.g., sample sizes (i.e., more/less Fe volume to prefill during incubation time before graphene nucleation), hydrocarbon type (with C_2_H_2_ fairly reactive) and desired growth times.^36,40^ The variation in growth time (Figure S4) indicates that graphene nucleation and growth begin within the first few minutes of exposure to the precursor, consistent with our *in situ* XPS data. Furthermore, we see that graphene domains merge quickly.

The carbon uptake into the Fe subsurface and bulk during graphene CVD is not only relevant for a more complete mechanistic understanding of graphene CVD on Fe but also has, via the concurrent surface hardening introduced here during graphene CVD, technologically beneficial implications. In particular, it shows the potential for the development of a combined graphene growth and surface hardening process for metallurgical materials, which is an aspect that has been addressed relatively little in the literature.

Another general finding pertaining to Fe is the here reported importance of controlling persistent Fe-surface oxides that can inhibit graphene growth and are harder to remove than in typical Ni- or Cu-based graphene recipes (which we can use without signs of persistent oxides on Ni or Cu in our CVD system^3,60^ ). We show, however, that also aided by the carbothermal reduction implicated here during hydrocarbon exposure, simple and cheap, scalable medium vacuum conditions, as used in this study, are sufficient to account for this higher propensity of Fe-oxide formation compared to other typical graphene growth substrates.

We anticipate the process to behave similarly when applied to steels, although the effect of the multiple additive alloying elements in modern steels will require further consideration individually. For low-carbon steel with minimal alloying elements, the process should be applicable without substantial changes, although slight parameter adjustments might be necessary to account for the dissolved carbon already present in the substrate before CVD. For high-alloy steel such as stainless steel, the constituent elements, in particular chrome (Cr), might require additional strategies to mitigate oxide formation during CVD. In general, this work lays a foundation for the successful growth of graphene via CVD for industrially relevant steel substrates by elucidating the controlling mechanisms for the pure Fe parent material.

We finally discuss a key limitation of our current study: our *in situ*-guided approach enabled and elucidated graphene growth results that go beyond prior graphene CVD on Fe with respect to quality and monolayer coverage under scalable CVD conditions.^21–35^ Nevertheless, the still remaining incomplete coverage of our graphene films (i.e., ∼20% bare substrate, as discussed above) hinders final large-scale application performance assessment as, e.g., macroscopic barrier layer or electrochemical corrosion protection.^67^ Nevertheless, to assess its protective capability at least on a local scale, we performed dry corrosion experiments by annealing the graphene-coated Fe foils on a hot plate in air.^68^ As shown in Figure S13, optical microscopy and Raman spectroscopy reveal that, where graphene is present, the graphene locally protects the Fe surface from significant oxidation during prolonged heating in air, compared to bare Fe areas that get strongly oxidized. This is a further indication of the high structural quality of the graphene grown and reaffirms that not the here produced structural graphene quality but rather achieving higher graphene coverage is the remaining key challenge to tackle in future work to move toward macroscopic performance assessment of our CVD graphene films on Fe in the future.

## Conclusions

In summary, we have developed a CVD process for the growth of graphene on iron substrates that can produce high-quality monolayer graphene films with a monolayer coverage of ∼70% and total graphene coverage of ∼80% under scalable CVD conditions. This represents a sizable improvement of graphene CVD on Fe and is a prerequisite for growing graphene on more complex multielement iron alloys such as steels. To obtain direct insights into the underlying growth mechanisms, we have followed the entire graphene CVD process on Fe using complementary *in situ* techniques to probe bulk crystallographic (*in situ* XRD) and surface chemical (*in situ* NAP XPS) evolution during CVD. Using this approach, we identified that specifically (i) carbothermal reduction of persistent Fe-oxides and (ii) kinetic balancing of carbon uptake into the Fe during CVD near the Fe–C eutectoid are critical for high-quality monolayer graphene CVD. Furthermore, we demonstrated that the carbon diffusion into the Fe is not only interesting from a growth mechanistic point of view but also akin to industrial surface hardening processes (carburization/case hardening) and, as such, can be beneficially utilized for establishing concurrent graphene CVD and surface hardening processes, as we also here demonstrated.

## Methods

### Graphene CVD

We employ a custom-built hot-wall CVD setup with a commercial split-tube furnace (Carbolite Gero Split Tube Furnace, HZS 12/600) around a quartz tube (GVB, EN08NB) for heating and temperature control and a combined, small turbomolecular pump/rotary vane pump stage (turbomolecular: VARIAN, Turbo-V 70LP, rotary: Vacuubrand RZ 2.5). In this configuration the base pressure of the CVD system is ∼3 × 10^–3^ mbar. For a typical CVD run, samples undergo annealing in ∼1 mbar H_2_ (∼250 sccm, Messer, 6.0 purity, 99.9999%) at the respective growth temperature (500 to 800 °C) for 30 min, after which 0.1 to 10 sccm of the carbon precursor acetylene (C_2_H_2_, Messer 2.6, 99.6% purity) is added for typically another 30 min. The samples are then left to cool naturally in ∼1 mbar of H_2_ with the split-tube furnace heaters opened around the quartz tube. H_2_ flow is controlled by a manual flow meter (Vögtlin Instruments GmbH, Q-Flow series) while C_2_H_2_ flow is controlled by a digital mass flow controller (MFC, Bronkhorst EL-flow select). We use 100 μm thick (Alfa Aesar Puratonic 99.995%) polycrystalline iron foils as a catalytic growth substrate. The Fe substrate is used as-received and is not subjected to any preprocessing or cleaning steps.

### *Ex Situ* Characterization

Samples are characterized *ex situ* via optical microscopy and Raman spectroscopy (WITec alpha 300 RSA+) after CVD. Laser wavelength 532 nm, laser power 10 mW, spot size ∼2 μm. Figure S14 shows consistent monolayer quality and coverage over the whole centimeter-scale substrate via optical and point-localized Raman spectra. Substrate “backside” for current optimal growth conditions was also characterized via optical microscopy and point-localized Raman spectroscopy, showing little to no difference in growth result compared to commonly examined “frontside”, shown in Figure S15.^69^ *Ex situ* XRD measurements were conducted with a PANalytical X́Pert Pro multipurpose diffractometer (MPD) with a standard rotating stage and chromium (Cr) anode as X-ray source with a wavelength of 2.26 Å. Presented Cr anode *ex situ* XRD patterns were scaled to make them comparable to the *in situ* XRD Cu anode data sets. While most characterization investigated the graphene growth results directly on their Fe growth substrates, for selected samples graphene film transfer^60^ was done using a poly­(methyl methacrylate)/ethyl acetate mixture for drop casting a sacrificial polymer layer on top the graphene/Fe foil sample, followed by a bubbling transfer procedure,^70^ before transferring the film onto a SiO_2_(90 nm)/Si substrate and dissolving the PMMA layer in acetone. For the bubbling transfer, the PMMA coated graphene/Fe foil is dipped into 0.5 molar K_2_SO_4_ together with a glassy carbon electrode. A voltage of about 4–5 V is applied with the glassy carbon electrode acting as the anode and the graphene/Fe foil acting as the cathode. Hydrogen bubbles are formed between the iron foil and the PMMA supported graphene, separating the graphene from the substrate. Transferred graphene is characterized by Raman spectroscopy^58^ and optical contrast analysis following a previously reported method.^71^ Graphene coverage was calculated using visual measurements (thresholding of optical microscopy image) of graphene films on Fe and a transferred graphene film on a SiO_2_(90 nm)/Si substrate. For the transferred films, coverage is potentially underestimated due to damage to the film during transfer.

### *In Situ* XRD

*In situ* XRD patterns were recorded on a PANalytical X́Pert Pro multipurpose diffractometer (MPD) in Bragg–Brentano geometry outfitted with an environmental cold-wall heating chamber (Anton Paar HTK 1200N) that can indirectly heat samples via a heating wire to up to ∼1200 °C and features atmospheric control through gas and vacuum regulation. Samples were placed on a ceramic sample holder, and temperature was monitored via a RhPt thermo couple. The anode material used as X-ray source was copper (Cu) for the *in situ* XRD, emitting Cu Kα_1_ and Cu Kα_2_ radiation (ratio 2:1) with a wavelength of 1.5406 Å. The 2θ range was set between 15° and 100°, and a scan rate of 4° min^–1^ was applied. H_2_ and C_2_H_2_ were fed via MFCs (Bronkhorst EL-flow select). Applied CVD conditions were similar to those in the hot-wall furnace system. Pumping employed a combined small turbomolecular pump/rotary value pump stage (turbomolecular: Oerlikon Leybold Vacuum Turbovac T50, rotary).

### *In Situ* NAP XPS

*In situ* NAP XPS experiments were performed at the CAT laboratory branches of the EMIL beamlines, UE48/PGM and CPMU17_EMIL, located at the synchrotron radiation facility BESSY II (Berlin, Germany). Applied CVD conditions were similar to those in the hot-wall furnace system, although the NAP XPS reaction chamber has a base pressure of ∼10^–8^ mbar. The focus points of both beamlines meet in a dedicated NAP XPS analysis system based on a SPECS Phoibos 150 analyzer which covers the kinetic energy range up to 7 keV. The UHV system is described in detail elsewhere.^72^ The sample temperature was measured through on surface clamped thermocouples, which however results in relatively large uncertainties for the Fe foils during graphene CVD (estimated to be ±100 °C). Thus, stated sample temperatures were also corrected against *ex situ* growth results.^43^ All XP spectra were recorded in normal photoemission geometry with a probing area of ∼60 μm × 120 μm corresponding to the profile of the incident X-ray beam. The overall spectral resolution of the NAP XPS system is about 0.4–0.5 eV at a 10 eV pass energy. The binding energy (BE) was calibrated using the valence band onset of metallic Fe with a pronounced Fermi edge with an accuracy of around 0.05 eV. In order to get an overview of the sample, survey spectra were recorded using a 1250 eV photon energy. Fe 2p, O 1s, and C 1s core levels were measured with 1250, 1050, and 800 eV, respectively. Details on the XPS data analysis are given in the Supporting Information.

### ToF-SIMS

ToF-SIMS was performed using a ToF-SIMS 5 instrument (IONTOF GmbH, Münster, Germany) equipped with a BiMn alloy liquid metal ion gun (LMIG), a dual-source column sputter gun (DSC), and an electron floodgun for charge compensation. A focused 25 keV Bi^+^ primary ion beam was employed to generate secondary ions, covering a mass range of *m*/*z* 1 to 230 (corresponding to a cycle time of 50 μs) in an analysis area of 100 × 100 μm. Measurements were carried out using sawtooth scanning with a pulse length of 13 ns in the high current bunched (HCBU) mode with a resolution of 128 × 128 pixels. This measurement mode provides high mass resolution (∼11,000) and a pulsed LMIG current of ∼2 pA. A 2 keV Cs^+^ ion beam (300 × 300 μm, ∼160 nA) was used for depth profiling since negative secondary ions like C^–^ are detected more sensitively with Cs^+^ bombardement.^73,74^ To ensure charge compensation during depth profiling, an electron flood gun set at 21 V was activated in a noninterlaced cycle mode (5 s sputtering; 0.5 s pause). Samples were introduced into the instrument and allowed to equilibrate overnight, stabilizing the chamber pressure at ∼5 × 10^–9^ mbar. The IONTOF ToF-SIMS instrument software, SurfaceLab 7 (version 7.1.130060), was used for data processing and mass calibration. The depth of the analysis craters was measured with a DekTakXT profilometer (Bruker) to convert the sputter time into depth.

### Hot Plate Oxidation

For the dry corrosion experiments, one H_2_-annealed Fe foil and one graphene-coated Fe foil were placed on a hot plate at 170 °C for 1 h 20 min in air.^68^ The annealed Fe foil underwent the same CVD thermal treatment without hydrocarbon exposure (750 °C, 60 min of annealing), while the graphene-coated sample was grown under identical conditions with 30 min of annealing followed by 30 min of exposure to 1 sccm of C_2_H_2_.

### Nanoindentation

Nanoindentation tests were carried out in a UMIS II nanoindentation system quipped with a Berkovich tip. Due to the rather thin samples, the load range was chosen to be 2–10 mN. Indents were made in steps of 0.5 mN. The recorded load–displacement curves were analyzed using the procedure described by Oliver and Pharr.^75^

## Supplementary Material



## Notes

Additional data and analysis including Raman spectroscopy, optical microscopy, XRD, XPS, and SIMS data (PDF)
