# Supplementary material for: Realizing Scalable Chemical Vapor Deposition of Monolayer Graphene Films on Iron with Concurrent Surface Hardening by In Situ Observations
Source: ACS Appl Mater Interfaces. 2026 Jan 28;18(5):8567–79. doi: 10.1021/acsami.5c18706 (PMC12903068; doi:10.1021/acsami.5c18706)
Supplement: Supplementary file 1 [file am5c18706_si_001.pdf]

Supporting Information to:

Realizing Scalable Chemical Vapor Deposition of Monolayer Graphene Films on Iron with Concurrent Surface Hardening by *in situ* Observations

Bernhard Fickl,[1] Werner Artner,[2] Daniel Matulka,[1,2] Jakob Rath,[1,3] Martin Nastran,[1] Markus Hofer,[1] Raoul Blume,[4] Michael Hävecker,[4,5] Alexander Kirnbauer,[6] Florian Fahrnberger,[7] Herbert Hutter,[7] Dengsong Zhang,[8,] Paul H. Mayrhofer,[6] Axel Knop-Gericke,[4,5] Beatriz Roldan Cuenya,[5] Robert Schlögl,[4,5] Christian Dipolt,[9] Dominik Eder,[1,*] Bernhard C. Bayer[1,*]*

[1]Institute of Materials Chemistry, Technische Universität Wien (TU Wien), Getreidemarkt 9, 1060 Vienna, Austria

[2]X-Ray Center, Technische Universität Wien (TU Wien), Getreidemarkt 9, 1060 Vienna, Austria

[3]Analytical Instrumentation Center (AIC), Technische Universität Wien (TU Wien), Lehargasse 6, 1060 Vienna, Austria

[4]Max-Planck-Institut für Chemische Energiekonversion, Postfach 101365, Mülheim an der Ruhr 45413, Germany

[5]Department of Inorganic Chemistry, Fritz-Haber-Institut der Max-Planck Gesellschaft, Faradayweg 4-6, 14195 Berlin, Germany

[6]Institute of Materials Science and Technology, Technische Universität Wien (TU Wien), Getreidemarkt 9, 1060 Vienna, Austria

[7]Institute of Chemical Technologies and Analytics, Technische Universität Wien (TU Wien), Getreidemarkt 9, 1060 Vienna, Austria

[8]International Joint Laboratory of Catalytic Chemistry, State Key Laboratory of Advanced Special Steel, Innovation Institute of Carbon Neutrality, Research Center of Nanoscience and Technology, Department of Chemistry, College of Sciences, Shanghai University, No. 99 Shangda Road; Shanghai 200444, China

[9]Rübig GmbH & Co KG, Schafwiesenstraße 56, 4600 Wels, Austria

*Corresponding Authors: bernhard.bayer-skoff@tuwien.ac.at (Bernhard C. Bayer), dominik.eder@tuwien.ac.at (Dominik Eder), dszhang@shu.edu.cn (Dengsong Zhang)

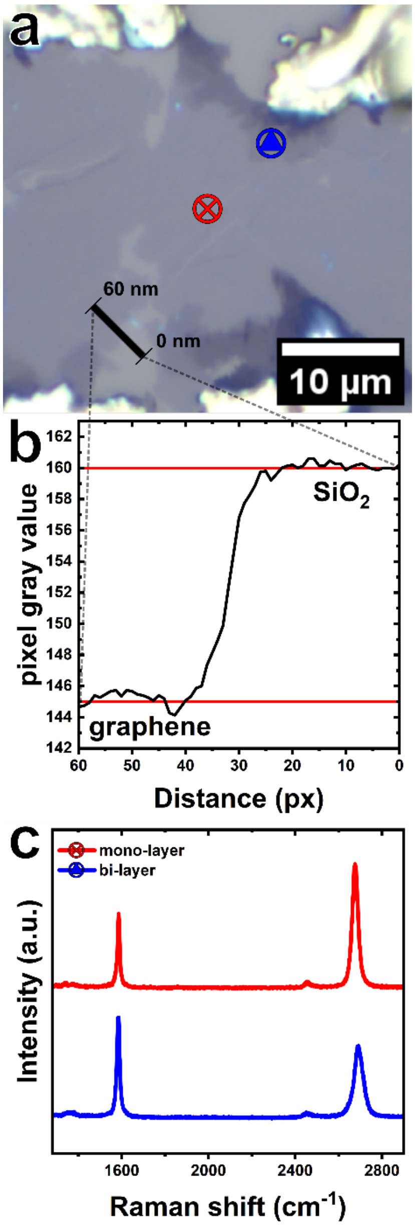

Supporting Figure S1. (a) Optical micrograph of CVD graphene from 750 °C/1 sccm C_2H_2 run from Figure 2 after polymer-assisted graphene transfer to 90 nm SiO_2-covered Si wafer. (b) Corresponding optical microscope pixel grey values averaged along the indicated black line in (a). (c) Corresponding point-localised Raman spectra with locations in (a) indicated by colour and symbol.

In Supporting Figure S1, we further characterise the optimised graphene films from the 750 °C and 1 sccm C_2H_2 runs in Figure 2. To this end, we transfer the films using a standard polymer-assisted transfer process[1] on 90 nm SiO_2-coated Si wafers. The optical images in Supporting Figure S1a, together with the Raman point-localised spectra (red trace) in Supporting Figure S1c, as well as the optical grey values in Supporting Figure S1b, reaffirm that the deposited film is predominantly monolayer graphene of high crystalline quality (D/G <3%).[2] The

monolayered nature of the graphene film is in particular confirmed by a 2D/G ratio of 2 and a 2D width of 28 cm^{-1} that is readily fitted with a single Lorentzian (thus excluding formation of turbostratic multilayer graphene), see Supporting Figure S2.[3] Furthermore, also the grey value in Supporting Figure S1b fully corresponds with the values expected for monolayer graphene.[4] In line with Figure 2, the remaining non-monolayer-graphene areas are isolated multilayer graphene islands (dark spots) and bare regions that we ascribe to prior Fe-oxide covered regions that did not nucleate graphene (plus losses of monolayer region from incomplete transfer).

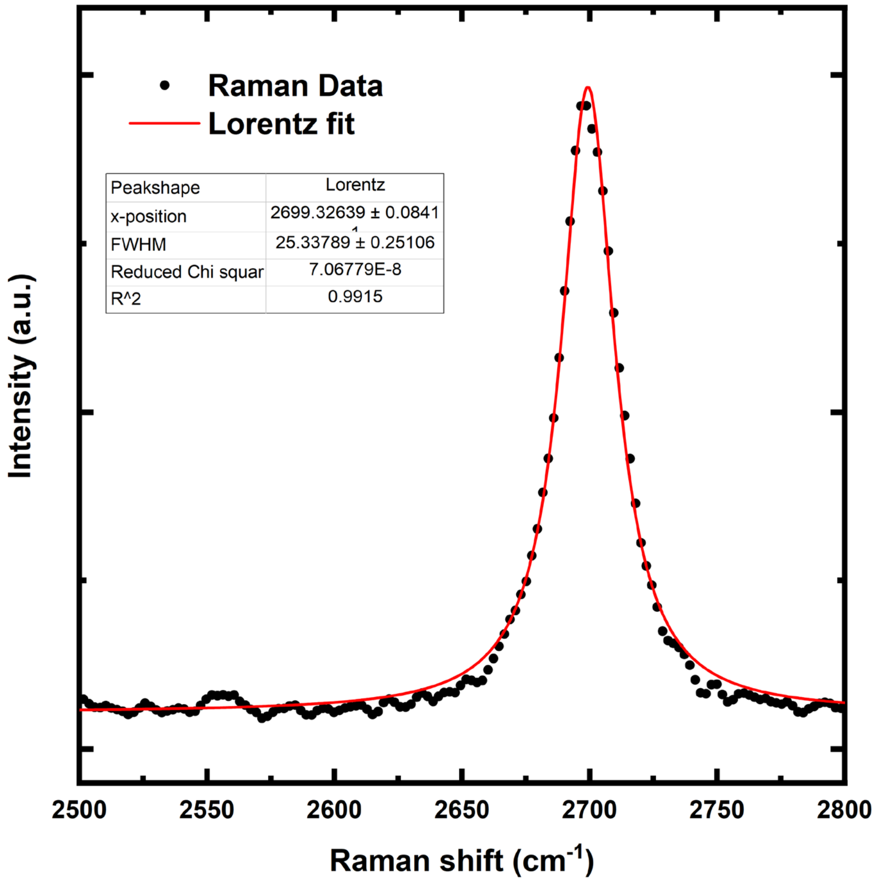

Supporting Figure S2. 2D peak of monolayer graphene film transferred from iron substrate onto SiO_2-coated Si wafer as in Supporting Figure S1 and fit with single Lorentzian (FWHM=25.3).[2]

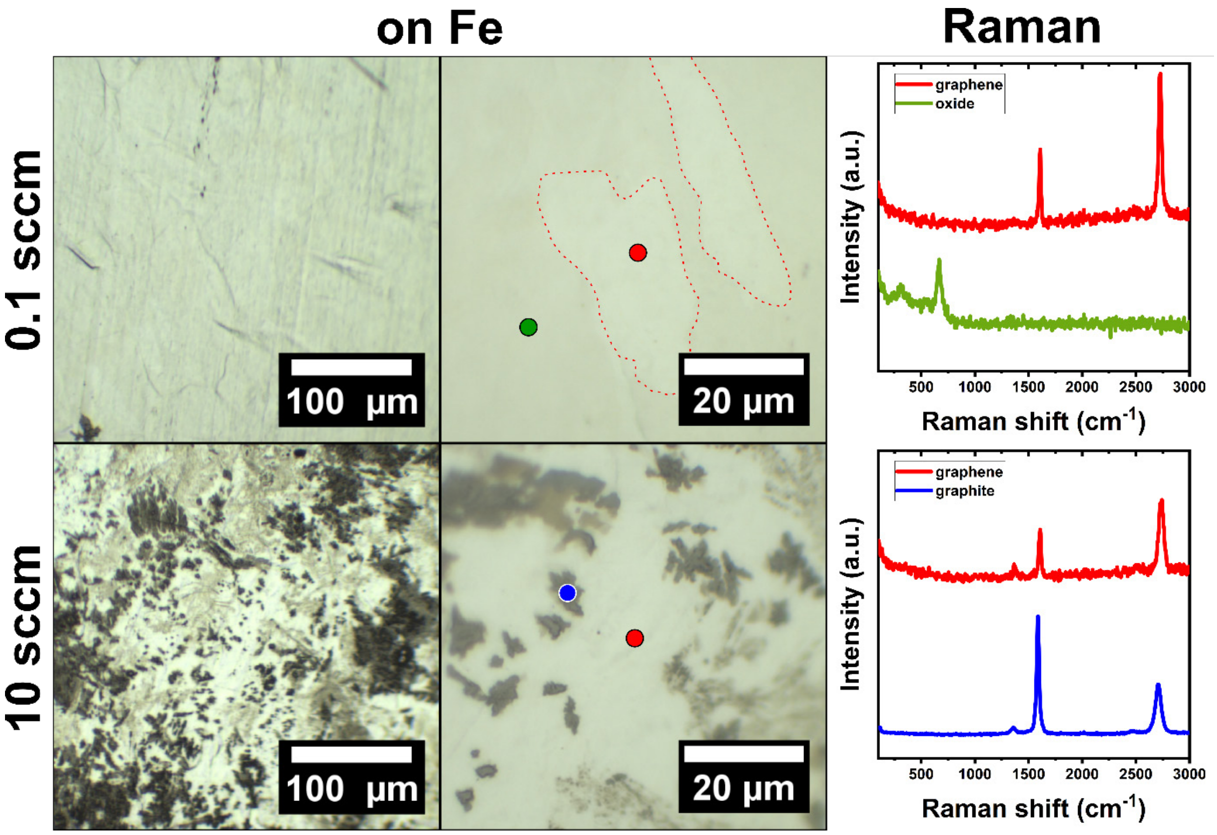

Supporting Figure S3. Optical micrographs (left and middle panels) at different magnifications and point-localised Raman spectra (right panels) for 0.1 sccm (upper panels) and 10 sccm (lower panels) of C_2H_2 precursor flow at 750 °C CVD temperature. High-quality monolayer graphene islands in the upper middle panel are indicated by dashed red outlines. The upper right panel shows Raman spectra of monolayer graphene regions (red) and iron oxide regions (green). The lower right panel shows Raman spectra of graphitic multilayer regions (blue) and monolayer graphene regions (red).

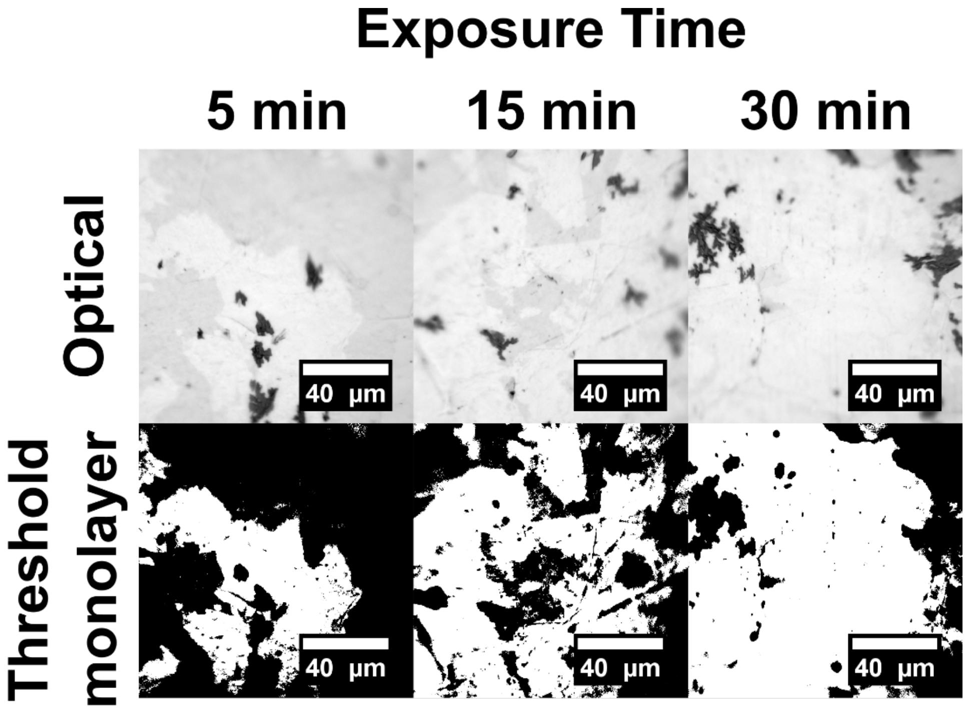

	5 min	15 min	30 min
Monolayer	~20%	~50%	~70%
Multilayer	~5%	~7%	~12%
no coverage	~75%	~43%	~18%

Supporting Figure S4. Optical images of Fe substrate surface after CVD graphene process at 750 °C with different C_2H_2 exposure times: standard 30 min, 15 min and 5 min. Below each optical image, a thresholded mask is displayed, showing the monolayer regions in white. Monolayer, multilayer and uncovered area fractions are shown below each image column. Area fraction values are averages of multiple sample spots and are subject to an error of around 10%. Error sources are inhomogeneous illumination of the optical microscope, local variations in coverage, as well as the general limitation of visual identification of coverage directly on the Fe substrate.

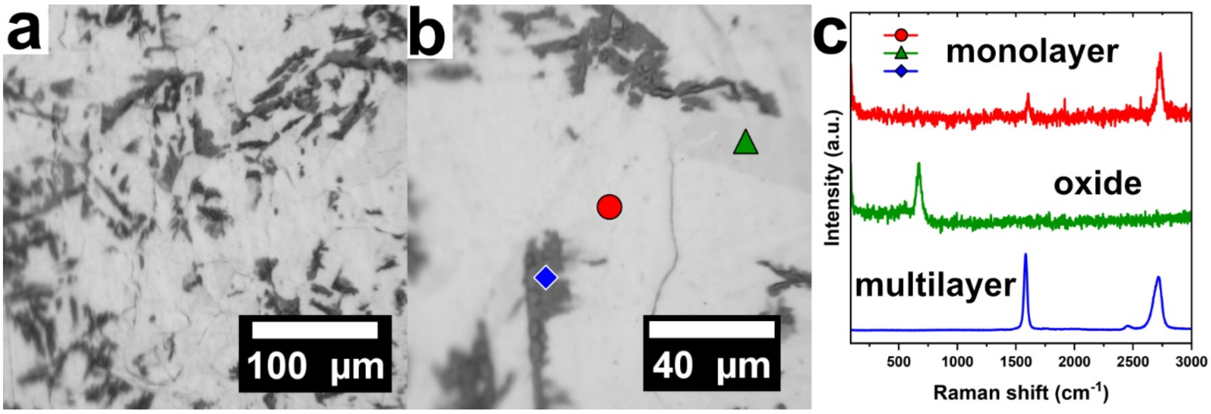

Supporting Figure S5. (a), (b) Optical micrographs of the Fe substrate after 60 min deposition at 750°C with 1 sccm C_2H_2, at 20× and 50× magnification, respectively. (c) Point localised Raman spectra showing monolayer graphene, Fe-oxide and multilayer Raman signals (points localisation indicated by coloured symbols in (b)). Average monolayer coverage ~52%, average multilayer coverage ~18%, average oxide coverage ~30%.

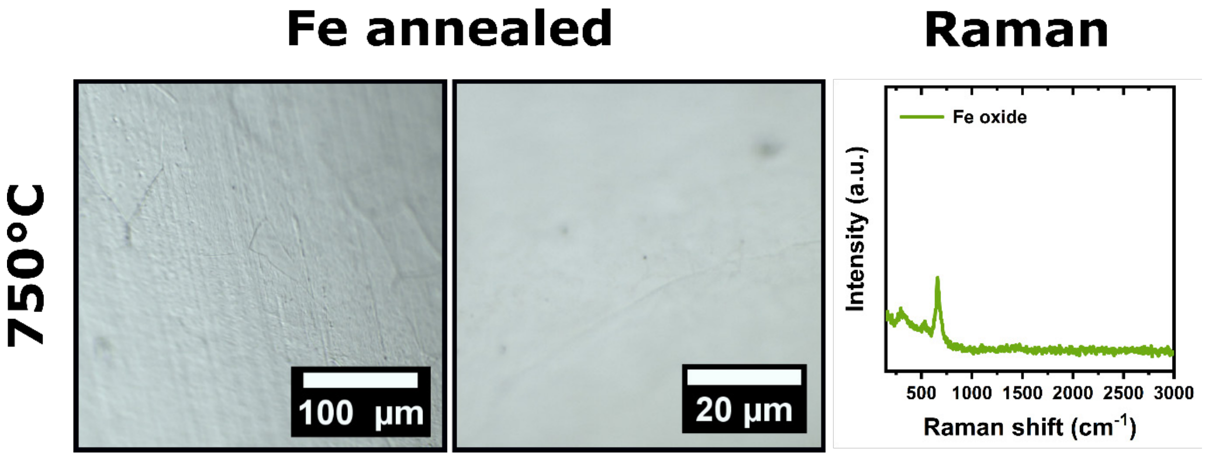

Supporting Figure S6. The left and middle panels show optical micrographs of H_2-annealed (750°C) Fe samples at different magnifications, respectively. The right panel shows a point-localised Raman spectrum and iron-oxide signal (green trace), representative of the entire sample surface.

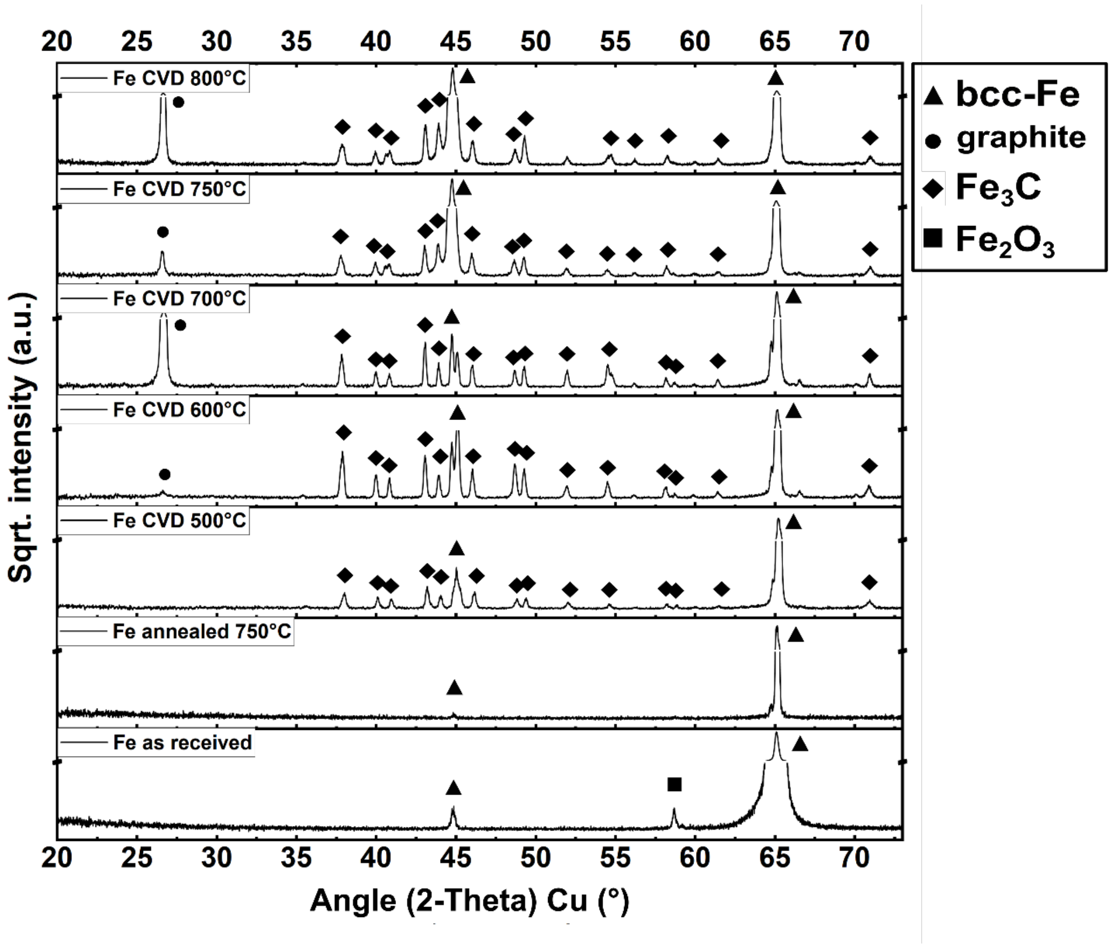

Supporting Figure S7. *Ex situ* XRD patterns of Fe catalysts supports after CVD conditions corresponding to Figure 2, as received Fe foil and after only H_2 treatment ("annealed at 750 °C"). Salient phases identified are indicated. (International Centre for Diffraction Data (ICDD), PDF-5+ database, powder diffraction file entry: bcc-Fe 04-015-8438; Carbon/graphite 04-016-0554; Fe_3C 04-007-0422) Note that the intensity scale is plotted in square-root and the intensity scale interruption(s) for better visualization of minor Fe_3C phase signal. Cr anode 2-Theta data was recalculated to Cu anode for better comparison.

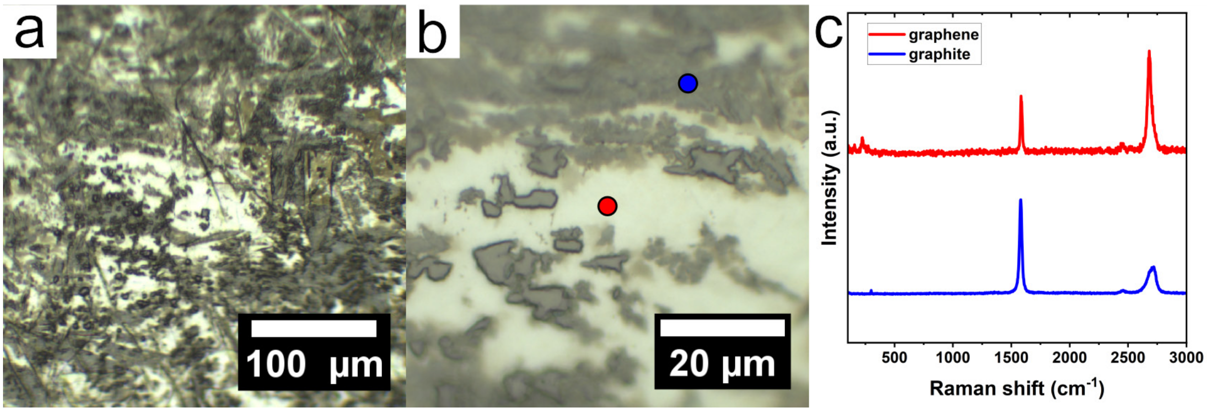

Supporting Figure S8. (a), (b) optical microscopy micrographs of the Fe surface after *in situ* XRD CVD process at ~750 °C, at 20× and 100× magnification, respectively. (c) Point-localised Raman spectra showing high quality, defect-free graphene and graphitic signals (points localisation indicated by coloured points in (b)).

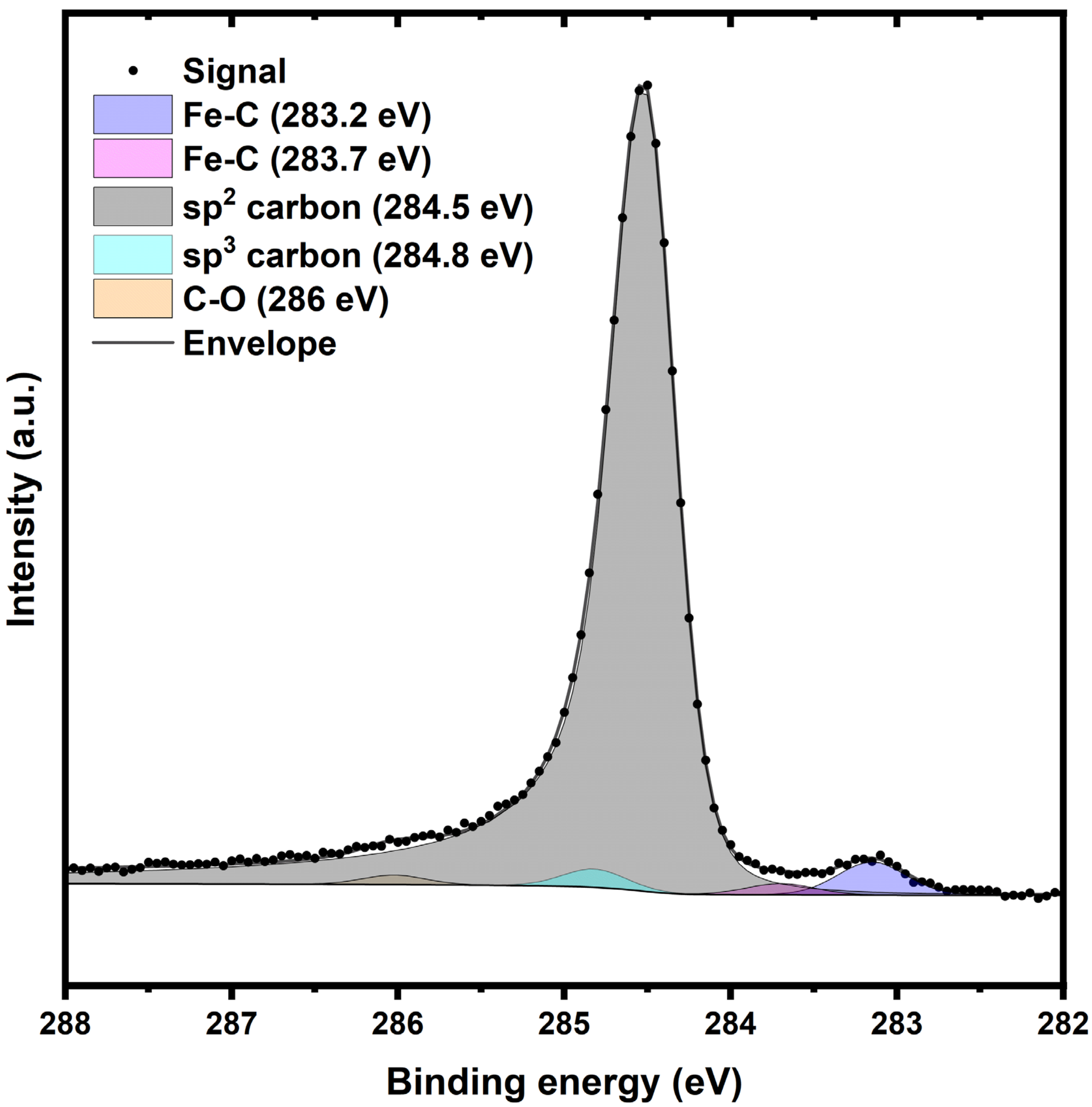

Supporting Figure S9. C1s spectrum during C_2H_2 exposure at 750 °C with peak components fit to experimental data, taken from the evolution in Figure 4a.

We identify in Supporting Figure S8 four primary components of our C1s peak related to graphene growth, plus one component considering the minimal contribution of C-O bound carbon. Firstly a peak at 283.2 eV which we ascribe to carbon bonded at iron surface sites based on previous work using Ni substrates.[5,6] On the same basis we identify a peak for carbon at interstitial Fe sites around 283.7 eV. We assign both components to carbon interacting with Fe and thus label both components “Fe-C”. We include a sp^2-hybridized carbon peak at 284.5 eV and a sp^3-hybridized carbon peak at around 284.8 eV (consistent with remaining defects, graphene edges and grain boundaries in the graphene) together with a C-O component at around 286 eV in accordance with literature.[7]

Peak fitting was done in CasaXPS software[8] and the peak shapes are given with the software command abbreviations. GL(30) denoting a Gaussian/Lorentzian product with 30% Lorentzian contribution. LF denotes an asymmetric Lorentzian lineshape with a tail dampening parameter.[9,10] Full-width-at-half-max (FWHM) of the sp^3 peak was constrained to the FWHM of the adventitious carbon peak, measured initially on bare Fe and the C-O component was constrained to follow the sp^3 component's FWHM. Fe-C components were also constrained to the same FWHM based on the FWHM of the peak at 283.2 eV. General peak shapes were based on work from Gengerbach et al..[7]

The XPS data in Supporting Figure S8 is consistent with high-quality graphene, reaffirming the Raman analysis in Supporting Figures S1 and S2.[11,12]

Peak designation	Peak position	Peak shape	FWHM
Fe-C	283.2 eV ± 0.1 eV	GL(30)	0.48 eV
Fe-C	283.7 eV ± 0.1 eV	GL(30)	0.48 eV
sp^2	284.5 eV ± 0.1 eV	LF(0.65,1.1,500,180,3)	0.45 eV
sp^3	284.8 eV ± 0.1 eV	GL(30)	0.69 eV
C-O	286 eV ± 0.1 eV	GL(30)	0.69 eV

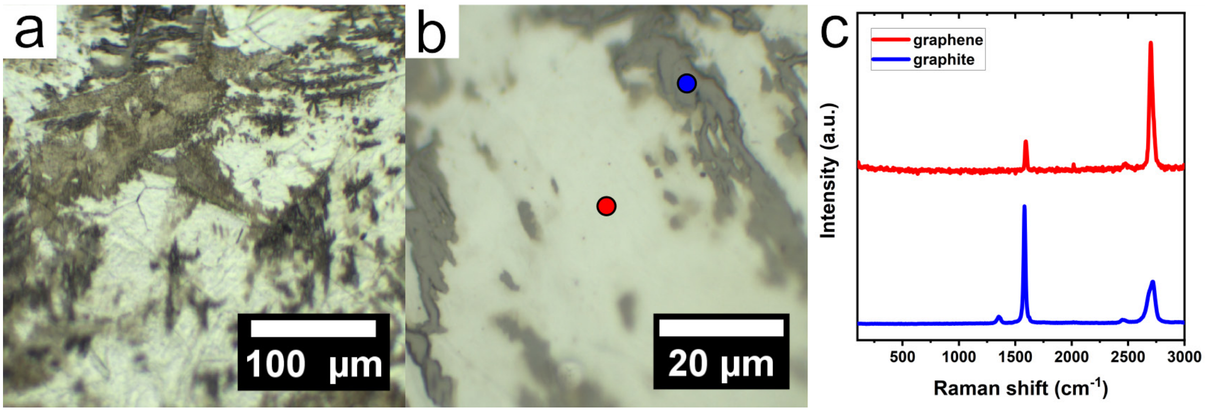

Supporting Figure S10. (a), (b) optical microscopy micrographs of the Fe surface after *in situ* NAP XPS CVD process at 750 °C, at 20× and 100× magnification, respectively. (c) Point-localised Raman spectra showing high quality, defect-free graphene and graphitic signals (points localisation indicated by coloured points in (b)).

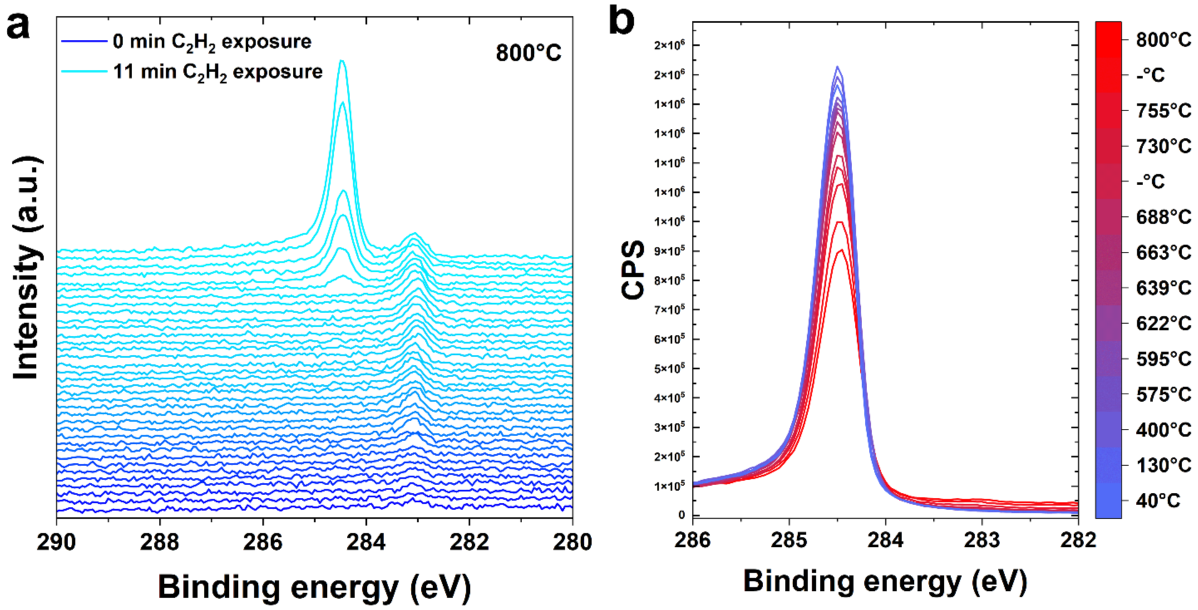

Supporting Figure S11. (a) Time-resolved in-situ XPS C1s spectra during C_2H_2 exposure at 800°C showing an incubation of ~10 minutes from hydrocarbon exposure start to start of isothermal surface carbon growth. Time increasing from 0 s to 11 min of exposure from bottom to top. (b) C1s time-resolved spectra during substrate cooling in H_2 atmosphere from 800 °C after C_2H_2 shut off show significant surface carbon intensity increase over time, reaching a plateau at around 400°C.

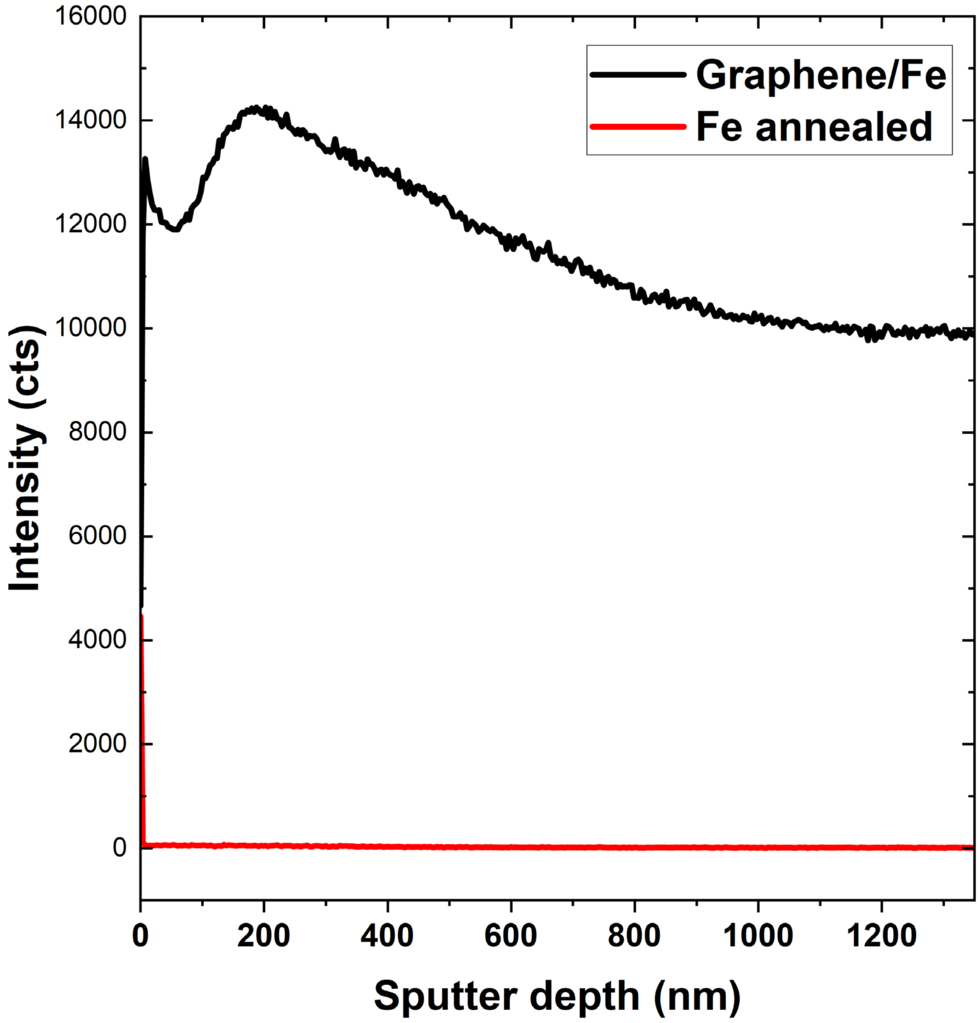

Supporting Figure S12. ToF-SIMS carbon anion C^- depth profile of H_2 annealed (without C_2H_2) reference Fe foil (red trace) and Fe foil after optimized CVD (black trace). The H_2 annealed Fe sample shows no significant carbon content. The CVD iron foil shows a large carbon signal and depth-dependent decrease, indicating a carbon diffusion gradient.

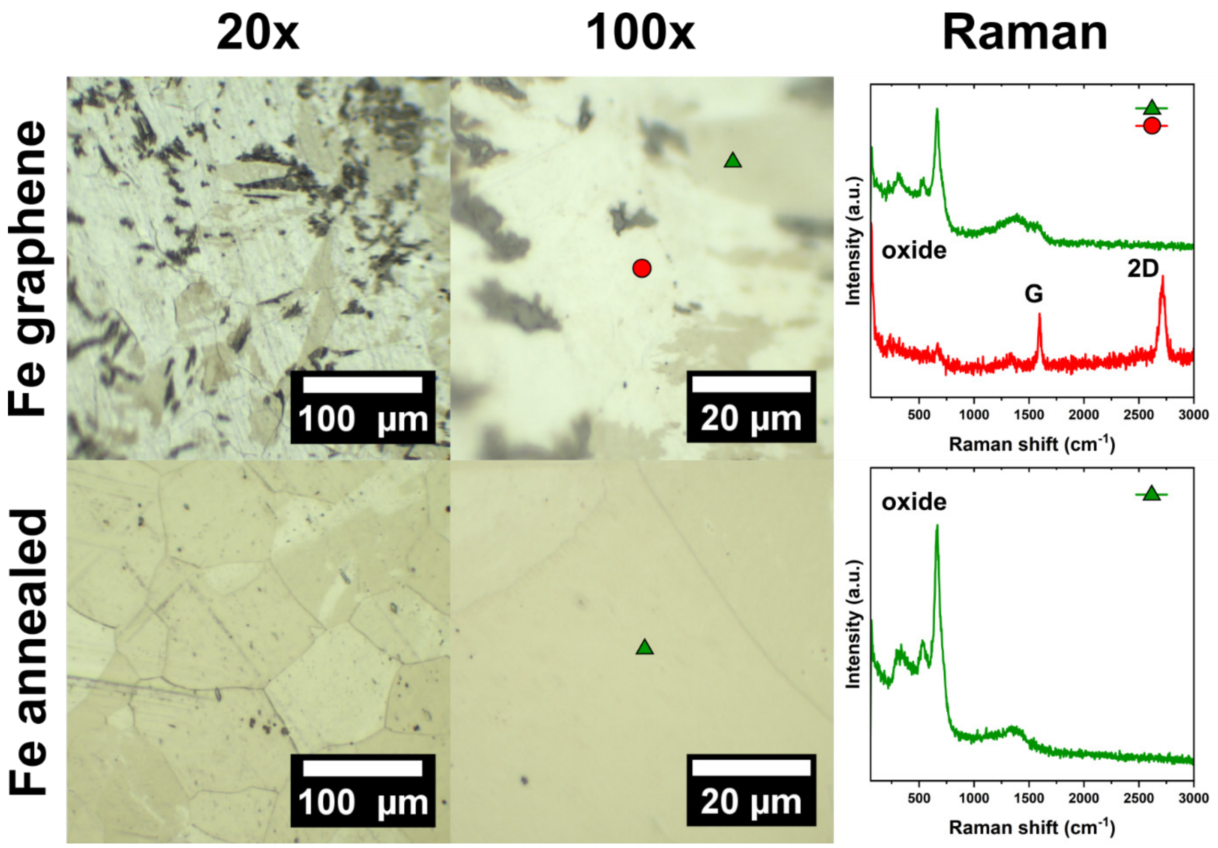

Supporting Figure S13. Optical micrographs (left and middle panels) at different magnifications and point localised Raman spectra (right panels) after hot plate oxidation[13] of graphene-covered (upper panels) and H_2-annealed (lower panels) Fe foils. The upper right panel shows Raman spectra of monolayer graphene regions (red) and iron oxide regions (green). The lower right panel shows Raman spectra of iron oxide regions (green), which are respresentative for the entire bare Fe reference sample.

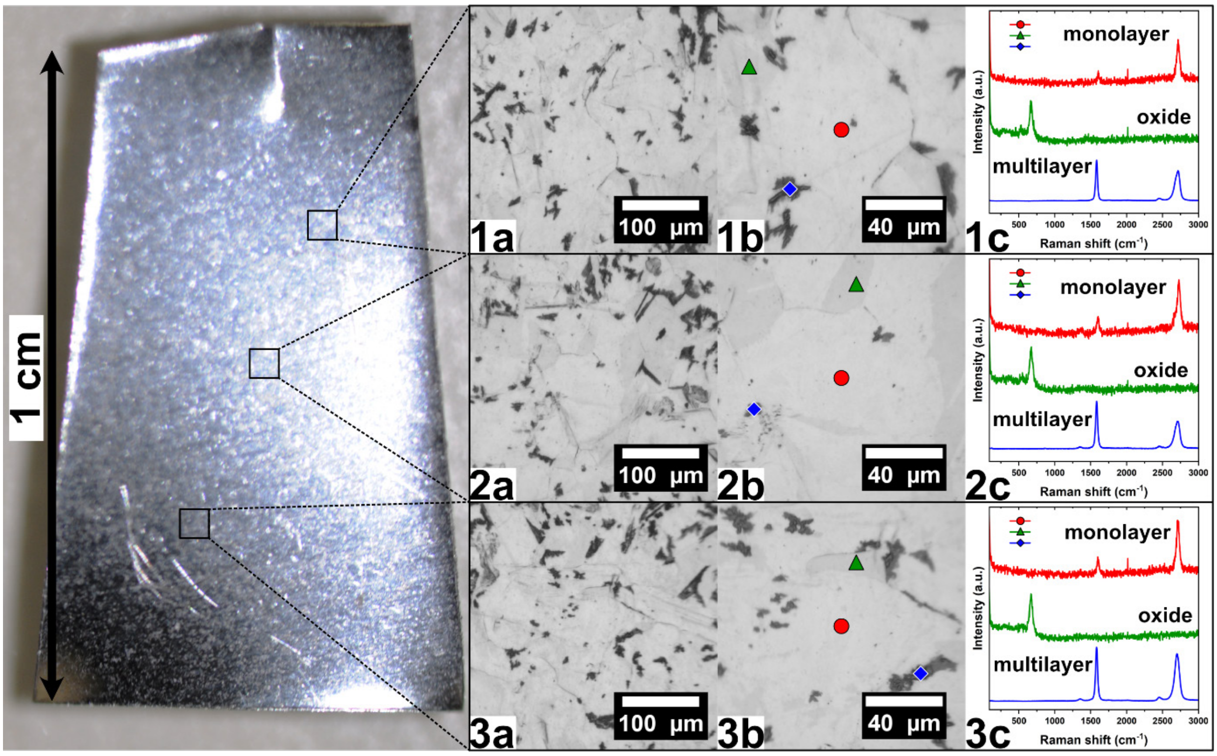

Supporting Figure S14. Optical micrographs (1-3, a-b) at three locations, indicated with black frames in the leftmost image, on the cm-scale Fe substrate after a standard CVD process. (1-3, c) Point localised Raman spectra of all three locations showing monolayer, oxide and multilayer regions indicated with symbols in the respective optical micrographs (1-3, b). Monolayer coverage of the measured regions is 1: ~72%, 2: ~67%, 3: ~68%.

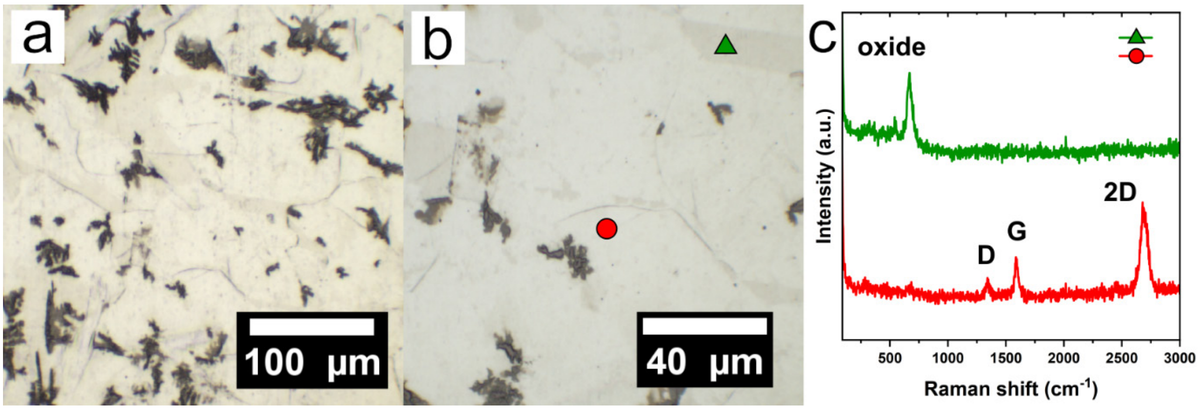

Supporting Figure S15. (a), (b) optical microscopy micrographs of the Fe substrate "backside", at 20× and 50× magnification, respectively. (c) Point-localised Raman spectra showing graphene and Fe-oxide Raman signals (points localisation indicated by coloured points in (b)).

Supporting References

(1) Fuchs, D.; Bayer, B. C.; Gupta, T.; Szabo, G. L.; Wilhelm, R. A.; Eder, D.; Meyer, J. C.; Steiner, S.; Gollas, B. Electrochemical Behavior of Graphene in a Deep Eutectic Solvent. *ACS Appl. Mater. Interfaces* **2020**, *12*, 40937–40948.
(2) Ferrari, A. C. Raman Spectroscopy of Graphene and Graphite: Disorder, Electron–Phonon Coupling, Doping and Nonadiabatic Effects. *Solid State Communications* **2007**, *143*, 47–57.
(3) Ferrari, A. C.; Meyer, J. C.; Scardaci, V.; Casiraghi, C.; Lazzeri, M.; Mauri, F.; Piscanec, S.; Jiang, D.; Novoselov, K. S.; Roth, S.; Geim, A. K. Raman Spectrum of Graphene and Graphene Layers. *Physical Review Letters* **2006**, *97*, 1–4.
(4) Blake, P.; Hill, E. W.; Castro Neto, A. H.; Novoselov, K. S.; Jiang, D.; Yang, R.; Booth, T. J.; Geim, A. K. Making Graphene Visible. *Applied Physics Letters* **2007**, *91*, 063124.
(5) Weatherup, R. S.; Bayer, B. C.; Blume, R.; Baehtz, C.; Kidambi, P. R.; Fouquet, M.; Wirth, C. T.; Schlögl, R.; Hofmann, S. On the Mechanisms of Ni-Catalysed Graphene Chemical Vapour Deposition. *ChemPhysChem* **2012**, *13*, 2544–2549.
(6) Bleu, Y.; Barnier, V.; Christien, F.; Bourquard, F.; Loir, A.-S.; Garrelie, F.; Donnet, C. Dynamics of Carbon Diffusion and Segregation through Nickel Catalyst, Investigated by in-Situ XPS, during the Growth of Nitrogen-Doped Graphene. *Carbon* **2019**, *155*, 410–420.
(7) Gengenbach, T. R.; Major, G. H.; Linford, M. R.; Easton, C. D. Practical Guides for X-Ray Photoelectron Spectroscopy (XPS): Interpreting the Carbon 1s Spectrum. *Journal of Vacuum Science & Technology A* **2021**, *39*, 013204.
(8) CasaXPS, 1999.
(9) Fairely, N. CasaXPS Manual 2.3. 15. *Casa Software Ltd* **2009**, 1–177.
(10) CasaXPS Manual-LA_Lineshape http://www.casaxps.com/help_manual/manual_updates/LA_Lineshape.pdf.
(11) Blume, R.; Rosenthal, D.; Tessonnier, J.-P.; Li, H.; Knop-Gericke, A.; Schlögl, R. Characterizing Graphitic Carbon with X-Ray Photoelectron Spectroscopy: A Step-by-Step Approach. *ChemCatChem* **2015**, *7*, 2871–2881.
(12) Kidambi, P. R.; Bayer, B. C.; Blume, R.; Wang, Z.-J.; Baehtz, C.; Weatherup, R. S.; Willinger, M.-G.; Schloegl, R.; Hofmann, S. Observing Graphene Grow: Catalyst–Graphene Interactions during Scalable Graphene Growth on Polycrystalline Copper. *Nano Letters* **2013**, *13*, 4769–4778.
(13) Chen, S.; Brown, L.; Levendorf, M.; Cai, W.; Ju, S.-Y.; Edgeworth, J.; Li, X.; Magnuson, C. W.; Velamakanni, A.; Piner, R. D.; Kang, J.; Park, J.; Ruoff, R. S. Oxidation Resistance of Graphene-Coated Cu and Cu/Ni Alloy. *ACS Nano* **2011**, *5*, 1321–1327.
